# Oriented Carbon Nanostructures by Plasma Processing: Recent Advances and Future Challenges

**DOI:** 10.3390/mi9110565

**Published:** 2018-11-01

**Authors:** Neelakandan M. Santhosh, Gregor Filipič, Elena Tatarova, Oleg Baranov, Hiroki Kondo, Makoto Sekine, Masaru Hori, Kostya (Ken) Ostrikov, Uroš Cvelbar

**Affiliations:** 1 Jožef Stefan Institute, Jamova cesta 39, SI-1000 Ljubljana, Slovenia; Neelakandan.M.Santhosh@ijs.si (N.M.S.); gregor.filipic@ijs.si (G.F.); Oleg.Baranov@post.com (O.B.); 2 Jozef Stefan International Postgraduate School, Jamova cesta 39, SI-1000 Ljubljana, Slovenia; 3 Instituto de Plasmas e Fusão Nuclear, Instituto Superior Técnico, Universidade de Lisboa, 1049 Lisboa, Portugal; e.tatarova@tecnico.ulisboa.pt; 4 Plasma Laboratory, National Aerospace University, Kharkov, Ukraine; 5 Department of Electrical Engineering and Computer Science, Nagoya University, Furo-cho Chikusa-ku, Nagoya 464-8603, Japan; hkondo@nagoya-u.jp (H.K.); sekine@plasma.engg.nagoya-u.ac.jp (M.S.); hori@nuee.nagoya-u.ac.jp (M.H.); 6 School of Chemistry, Physics, and Mechanical Engineering, Queensland University of Technology QUT, Brisbane, Australia; kostya.ostrikov@qut.edu.au; 7 CSIRO-QUT Joint Sustainable Processes and Devices Laboratory, P.O. Box 218, Lindfield, NSW 2070, Australia

**Keywords:** carbon nanostructures, carbon nanowall, graphene nanowall, plasma-enhanced chemical vapor deposition

## Abstract

Carbon, one of the most abundant materials, is very attractive for many applications because it exists in a variety of forms based on dimensions, such as zero-dimensional (0D), one-dimensional (1D), two-dimensional (2D), and-three dimensional (3D). Carbon nanowall (CNW) is a vertically-oriented 2D form of a graphene-like structure with open boundaries, sharp edges, nonstacking morphology, large interlayer spacing, and a huge surface area. Plasma-enhanced chemical vapor deposition (PECVD) is widely used for the large-scale synthesis and functionalization of carbon nanowalls (CNWs) with different types of plasma activation. Plasma-enhanced techniques open up possibilities to improve the structure and morphology of CNWs by controlling the plasma discharge parameters. Plasma-assisted surface treatment on CNWs improves their stability against structural degradation and surface chemistry with enhanced electrical and chemical properties. These advantages broaden the applications of CNWs in electrochemical energy storage devices, catalysis, and electronic devices and sensing devices to extremely thin black body coatings. However, the controlled growth of CNWs for specific applications remains a challenge. In these aspects, this review discusses the growth of CNWs using different plasma activation, the influence of various plasma-discharge parameters, and plasma-assisted surface treatment techniques for tailoring the properties of CNWs. The challenges and possibilities of CNW-related research are also discussed.

## 1. Introduction

**Figure 1:**
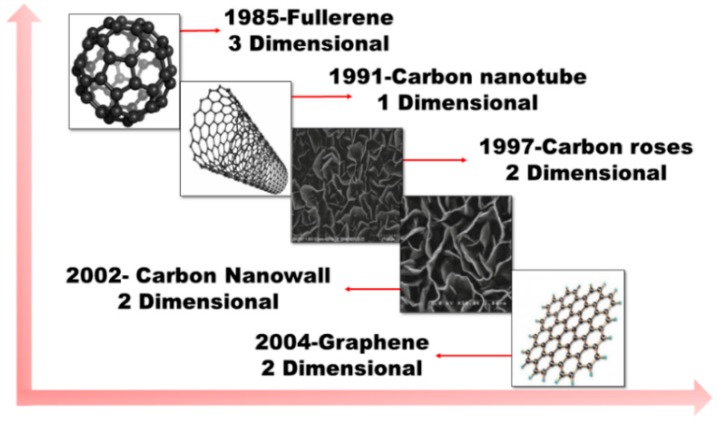
Milestones in carbon nanostructure research.

The unusual characteristic properties, from structural and morphological to electrical, of two-dimensional (2D) carbon nanostructures have made them an attractive material for a wide range of applications. Their investigation was started in the early 1980s after various carbon nanostructures were distinguished based on their dimension. Fullerene, which belongs to a zero-dimensional (0D) carbon nanostructure, was reported first [1], followed by one-dimensional (1D) carbon nanotubes (CNTs). Carbon roses were the first reported 2D carbon nanostructures [2]. Then, the first vertical sheet-like structures, that is, carbon nanowalls (CNWs)/graphene nanowalls (GNWs), were reported in 2002 [3]. However, the actual development of 2D material research started to bloom with the isolation of graphene in 2004 [4] and the later realization that 2D carbon nanomaterials are composed of graphene sheets in various compositions. The milestones in carbon nanostructure research are shown in Figure 1. The importance of graphene is that it is the building block of many carbon nanomaterials: Fullerene (0D) is graphene wrapped in a sphere, CNTs (1D) are graphene rolled into tubes, and CNWs are graphene sheets with open boundary and sharp edges normal to the substrate surface. All these structures can be single-layered or multilayered graphene sheets with different interlayer spacing. CNWs are self-assembled, vertically-oriented arrays of open boundary structured few graphene sheets, which are separated with an interlayer spacing of several nanometers and with a large surface area. Their height is in the range of 1–2 µm, with a thickness in the order of several nanometers. Good thermal and electrical characteristics with a high mechanical stability of CNWs make them an attractive material for a wide range of applications, such as catalyst supporters for fuel cells [5,6], catalytic activity towards oxygen reduction reaction [7], battery electrode materials [8,9,10], templates for fabrication of nanostructures [11,12,13], gas sensor materials [14], resistive switching memory devices [15], field emission devices [16,17,18], and superhydrophobic surfaces [19].

The conventional micromechanical exfoliation, chemical vapor deposition (CVD), and epitaxial growth techniques have been used for the synthesis of single- or multilayered graphene and other 2D graphene forms [20]. However, none of these techniques are assured regarding the structure quality, size control, and rate of growth; exfoliation techniques suffer from structure defects, the shape, and uncontrollability of size. Thermal CVD techniques enable the growth of high-quality 2D carbon nanostructures, but the synthesis is limited due to the need for very high temperatures in the range of 1000–1700 °C [21,22,23]. Epitaxial growth also requires a high temperature (1500 °C) for the growth process to attain the high-quality 2D carbon nanostructures [23]. This indicates that all these techniques are not able to supply a large-scale synthesis and processing of 2D carbon nanostructures which would be needed for industrial applications. Thus, improved techniques are needed. One of them could be a group of synthesis methods connected with reactive gaseous plasma. There are already many reports on plasma or plasma-assisted synthesis of 2D carbon nanostructures, including CNWs, few-layer graphene sheet (FLG), graphene, etc. [3,24,25,26,27,28] As different applications demand specific material properties, the way in which CNWs are synthesized matters greatly. Plasma-enhanced chemical vapor deposition (PECVD) has already been shown to deposit high-quality CNWs [29,30]. During PECVD, one can control the growth rate of CNWs by controlling the discharge parameters of plasma (gas selection, power density, substrate material and temperature selection, etc.) [31]. The ability to alter the physical and chemical properties of material simultaneously during deposition is one of the main advantages of plasma techniques. The properties can be modified by surface functionalization, exchange of atoms in the crystal structure (e.g., material doping), and defect density control. Compared to conventional chemical synthesis roots, plasma-based techniques also offer the large-scale synthesis of 2D carbon nanostructures [32].

Compared to other established synthesis techniques, plasma offers control over the growth of CNWs, with enhanced physical and chemical properties at large-scale. As such, this paper will try to describe the general principles of plasma-enhanced synthesis of oriented 2D carbon nanostructures and finding the influence of different discharge parameters on nanostructure growth. Furthermore, it will deal with plasma parameters and particles and present how this knowledge can be useful in plasma chemistry for the functionalization of CNWs, which can be done after synthesis or even during growth itself. In addition to functionalization, the study will also immerse into finding the advantage of plasma techniques for doping the CNWs. The review will conclude with identifications of challenges and possibilities of plasma-assisted CNW synthesis and their future applications.

## 2. Plasma: Potential Approach for Carbon Nanowall (CNW) Synthesis

Plasma synthesis of CNW can follow two paths: Either deposition of carbon species and followed growth of CNW or restructuring of carbon material and growth of CNW on top of it. The first one is plasma vapor deposition, and it is the most common; thus, the majority of this chapter will be about that. The general principle of PECVD techniques for CNW growth is the gas phase deposition process, where the carbon source gas is introduced into the plasma, where it gets chemically activated through partial ionization, dissociation, and even electron excitation. These radicals are then transported to the substrate at optimal condition, namely optimal substrate temperature, and the synthesis of nanostructured CNWs occurs.

Plasma is generated by the application of a strong electromagnetic field, which accelerates electrons to collide with the neutral gas. This leads to the dissociation, ionization, and excitation, which form numerous species—more electrons, ions, photons, and radicals, as well as excited background gas. Based on the electron temperature, plasma can be distinguished as high-temperature plasmas (above 10 eV) and low-temperature plasmas (<1 eV). Another criterion is based on the temperature which separates into equilibrium and nonequilibrium plasmas, where the temperature of ions is equal to the electron temperature in the former, and nonequal in the latter case. Plasmas can also be atmospheric or low-pressure (10^2^–10^4^ Pa). When plasma is used for material synthesis or processing, each of its particles play a significant role: (1) Energetic ions cause sputtering of material, elevate the substrate temperature, and increase surface diffusion; (2) electrons are involved in the chemical reactions by supplying activation energy; (3) photons which are generated during the de-excitation of the plasma molecules, atoms, and ions heat the substrate and take place in the photochemical reactions; (4) highly reactive radicals are deposited and react with the substrate or chemically etch the sample; (5) a fraction of the undissociated source gas is also involved in chemical reactions on the substrate [33]. Additionally, one can further influence ion and electron energy by biasing the sample and chemical reactions on the surface through external heating of the sample. These various factors make plasma a versatile tool for chemical synthesis, deposition, and surface treatment. The combination of plasma species–substrate interactions also enables a wide selection of substrates, since many chemical reactions and deposition processes can occur at much lower temperatures compared to, for example, thermal CVD, which occurs at a temperature above 1000 °C [34]. In this aspect, low-temperature and low-pressure PECVD techniques have emerged as an important method for the large-scale synthesis of CNWs.

**Figure 2:**
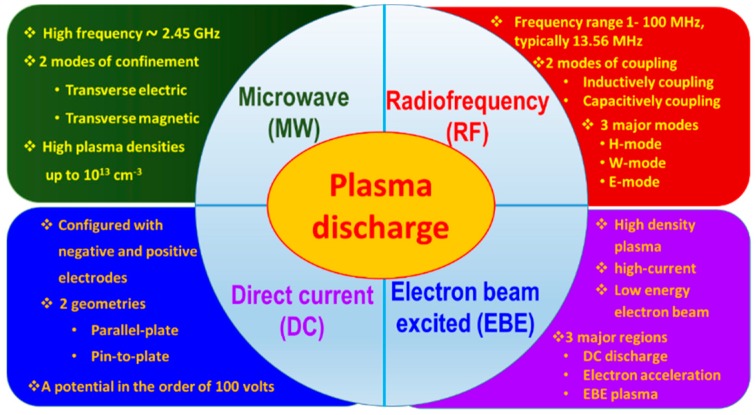
Different plasma systems for the carbon nanowall (CNW) growth.

A PECVD method is a system with typically low pressure to control the purity of the deposition. However, there can be different plasma sources, power supplies, and antenna combinations, which have different characteristic properties, and consequently, CNW depositions have different features. A PECVD system mainly consists of three major parts; they are (i) plasma generator (plasma source and antennas); (ii) gas (precursor and etchants); (iii) vacuum heating chamber, usually a quartz tube (substrate placed, and plasma interactions occur for the deposition). Based on the plasma generators, plasma sources, such as a microwave (MW), radio frequency (RF), direct current (DC), and their combinations, are widely used for CNW synthesis. Figure 2 shows the different plasma systems for the growth of CNWs based on the plasma sources. Microwave plasma-enhanced chemical vapor deposition (MWPECVD) is a high-frequency plasma system with a MW generator that has a frequency of approximately 2.45 GHz. The MW is generated from the MW source and coupled to the vacuum heating chamber either via a traverse rectangular cavity guide or using an external antenna, which produces a higher electric field effect inside the chamber. The wave propagation mode in the traverse rectangular cavity guide coupled MW system is transverse electric mode (TE). The electromagnetic waves in the waveguide interact with the plasma discharge to form a standing wave. An external tuner is used for controlling the waveguide length to attain the maximum electric field in the growth region. In TE mode, the electrons do not experience any change in the electric field and gain energy through the collision between background gases. The low dimension (wavelength) of the collimated MW drives the TE mode to attain the maximum electric field, which increases the electron-neutral collision and produces radicals for the deposition process. The TE–MWPECVD system is typically operated under 100–600 W. The high power may damage the quartz tube and vacuum system. The quasi-optical nature of MW made them difficult to confine and leads to diffraction in the tube, which results in the lower growth rate and non-uniformity in the morphology of CNWs. An external antenna is placed to the vacuum chamber used to couple the dominant cylindrical waveguide to the system through transverse magnetic (TM) mode wave propagation. This external antenna can increase the intensity of the electric field at the substrate surface placed inside the chamber in the form of a plasma ball. This plasma ball covers the substrate entirely and helps to control the substrate temperature and promotes the uniform growth of CNWs [29,35].

RF plasma systems are another type of AC plasma system, where RF waves with a frequency in the range of MHz (typically 13.56 MHz) are coupled to the plasma sources by two different antennae; they are inductively coupled plasma (ICP) and capacitively coupled plasma (CCP). The energy from the RF generator to the plasma system is coupled through these couplings in three modes, that is, evanescent electromagnetic mode (H), propagating wave mode (W), and electrostatic mode (E) [35]. In ICP systems, the energy from the RF generator is coupled through the inductive coils, which stimulates the magnetic field in the ICP discharges. Azarenkov et al. proposed that the discharge length and plasma densities in an RF system can be varied by changing the external magnetic field and keeping other plasma parameters fixed [36]. This magnetic field induces a low amount of high-frequency electric field for the ionization, which is suitable for operating under low pressure. Based on the geometry, different antennas used in an ICP system for the plasma discharge are distinguished into two types: Planar geometry and cylindrical geometry. In a planar geometry, a coil antenna of flat metal is used as the electrode. In cylindrical geometry, an inductive coil is surrounded on the quartz tube. Here, the inductive coil can produce partially longitudinal transverse electromagnetic waves by changing the magnetic field, which is coupled with evanescent electromagnetic waves (H-mode) to plasma. In helicon plasma systems, the coil is coupled to the RF generator through the helical spring-like structure, and a propagating wave (W-mode) helicon plasma is obtained by the effect of the magnetic field (100–300 G) [35]. A theoretical study to optimize the various antennas as a potential plasma source to produce IC plasma by Gogolides et al. stated that, the electric field was scale with the number of loops of the coil for a constant current [37]. Compared to the ICP systems, a CCP system consists of two parallel plate electrodes, where the electrostatic waves (E-mode) are produced in between these two parallel plate electrodes, which are capacitively coupled to the RF source for the plasma discharge. ICP plasmas have more advantages compared to the plasma produced by CCP mode, that is, they are capable of producing very high electron density and reactive species (up to approximately two orders of magnitude higher than the standard CCP plasmas under a similar plasma condition), self-regulating control of the ion energies due to the collision, low electron temperature, and plasma sheath potential, with uniform plasma discharge parameters along the radial and axial directions [38]. Due to the lower electron density and electron energy in CCP mode, successful growth of CNWs became hard using a capacitively coupled plasma-enhanced chemical vapor deposition (CCPECVD) system as an independent source [39,40]. Therefore, CCP mode is usually combined with other high-density plasmas for attaining a higher growth rate of CNWs. The combinations of different modes with CCP widely used for enhancing the CNW growth are: CCP+ICP, CCP+MW, and CCP+IC H_2_ [41,42]. In all these combinations, the carbon-containing gases are dissociated to form radicals by the electric field produced between parallel plate electrodes [42].

Compared to the two abovementioned plasma systems, plasma in a direct current (DC) system is produced by the glow discharge between two electrodes (cathode and anode) when DC passes through the gas in between the electrode. The DC glow discharge can be produced between the electrodes in two modes: (i) Parallel-plate and (ii) pin-to-plate. In a parallel plate DC discharge, a sufficient potential is applied between two parallel plate electrodes to break down the gas composition. The ionization of gas molecules increases by increasing the electric field between the electrodes, and the glow discharge is produced. A strong electric field and an ion flux between the electrodes enhance the growth rate and orientation of the CNWs. In an asymmetrical pin-to-plate electrode system, a sharp pin (typically tungsten) is attached to a planar electrode, and the high-intensity electric field is produced near the tip. This high electric field enhances the ionization rate of gas molecules and helps with the massive production of CNWs [14,43]. DC power sources are also used for the plasma discharge in electron beam excited plasma (EBEP) to achieve high-density plasma, where the plasma is produced by a high-current and low-energy electron beam. The electron current is controlled by the discharge current and electron beam energy is controlled by accelerating voltage. An electron beam excited plasma-enhanced chemical vapor deposition (EBEPECVD) system mainly consists of a DC plasma discharge region, electron acceleration region, and EBEP region. A cathode (usually LaB_6_) produces electrons and sustains the DC plasma to extract an electron beam. The electron beam causes the dissociation of gas molecules by providing sufficient energy. EBEP enables the production of highly ionized plasma at low pressures by adjusting electron beam energy close to the maximum electron impact ionization energy of the source gas. The higher density of source gas inside the EBEP system increases the pressure inside the chamber, which assists in controlling the morphology of CNWs [44,45]. In addition to these plasma-enhanced deposition techniques, several reports on the growth of carbon nanostructures and graphene flakes have been successfully demonstrated by arc discharge plasma [46,47]. The first reported petal-like 2D carbon nanostructures were also synthesized by arc discharge. However, the lack of available literature on the vertical growth of CNWs through arc discharge plasma shows that the focus on CNW growth through arc discharge plasma is not exploited well. All these plasma systems mentioned above imply that the large-scale production of CNWs is highly influenced by the density of plasma activated species. The effect of plasma-activated species on the growth of CNWs will be discussed in the forthcoming sections of this review.

A summary of the CNW depositions with different plasma sources and discharge parameters that were reported is in Table 1. The extensive studies on the growth of CNWs using different plasma sources reveal that the structure and morphology of CNWs can be altered by varying the gas proportion and gas flow rate. A higher flow of gas mixtures gives a higher growth rate of CNWs and higher rate of etching effect. However, a higher concentration of gases inside plasma systems increases the pressure inside the plasma system, which degrades the stability of plasma discharge and retards growth. Plasma surface interactions can heat the substrate surface to a certain extent, which also influences the morphology and structure of CNWs. However, the presence of energetic electrons and other active species in plasma keep the heat effects lower compared to thermal CVD. CNWs with a similar morphology can be synthesized by varying discharge parameters and plasma sources. On the other hand, similar plasma sources with different discharge conditions provide the opportunity to synthesize CNWs with a different morphology, interlayer spacing, height-uniformity, and thickness. For example, considering the works reported on MWPECVD-synthesized CNWs, the features of CNWs were changed by varying the flow rate, growth time, and temperature [3,25,26,48]. Alternatively, vertically-oriented CNWs with similar features were reported using two entirely different plasma systems; radio-frequency capacitively coupled plasma-enhanced chemical vapor deposition (RFCCPECVD) and direct current plasma-enhanced chemical vapor deposition (DCPECVD) and at different growth time [41,45,49]. CNWs with similar structural properties were reported using the same plasma source, the source gas, and plasma power but varying growth time and flow rate of gas [3,25]. On the other hand, CNWs with different morphologies were synthesized using different source gases and growth temperature [31,50]. In general, it was found that the morphology of CNWs was greatly influenced by the high density of carbon atoms, temperature, and pressure inside the plasma system. In all these cases, high-density MW, radio-frequency inductively coupled plasmas (RFICP), and DC plasmas are mainly used for the production of a large number of carbon dimers (C_2_ radicals) and hydrogen radicals for the deposition process. All the RFCCP systems are combined with either a high-density ICP system or external radical injections to achieve a large number of H radicals to remove the amorphous carbon (a-C) phase. Furthermore, in all cases, the substrate temperature is kept below 400–850 °C for the vertical growth of CNWs, which is much lower than the average temperature of the thermal CVD process (operational temperature 1200 °C).

Carbon-containing aromatic compounds were also successfully employed for the large-scale growth of CNWs. Lehmann et al. described the growth of CNWs using paraxylene as the precursor in an RF–ICP system [57]. The morphology of carbon nanostructure changes from nanofibers, nanowalls to interconnected nanowalls concerning the flow rate decreases from 5 mL/h to 0.5 mL/h. It was observed that the number of defects in the carbon nanostructures increased with the decrease in the flow rate. The interconnected CNWs contain atomic defects, mainly due to the twisting and bending of nanowalls in different directions, were formed at a low flow rate. Giese et al. reported the growth of CNWs from a single source metal organic precursor Al(acac)_3_ using an RFICP system [50]. The powdered form of the precursor was transported into the chamber using an evaporator bed with the assistance of Ar gas. The growth of CNWs was explained by the function of substrate bias and substrate temperature. The curled thin CNWs with the large surface area, higher wall height, and higher surface densities were deposited at the highest value of temperature and bias. The solid aromatic precursor provides an advantage to dop metallic nanoparticles in the CNW lattices with a uniform distribution. Bundaleska et al. reported the advantage of the aromatic precursor, containing functional groups to achieve CNW growth with a higher number of defects [58]. The mixture of ethanol and ammonia was used in an MW atmospheric condition for the growth and nitrogen doped CNWs were deposited on the substrate. Therefore, the growth of CNWs from aromatic precursors containing different functional groups would be a potential method to control the growth in the desired manner; with functional entities, uniform height distribution, etc.

An alternative plasma synthesis to PECVD is the direct surface growth of CNWs from a bulk carbon precursor using plasma-assisted carbonization. Ostrikov et al. reported a method for the growth of vertical graphene sheets (VGS) from natural honey [59]. A Si substrate was coated with liquefied honey and loaded in an RFICP system. An Ar:H_2_ gas mixture with a flow rate of 10:7 sccm was used for surface treatment on honey. After 10 min of carbonization, VGS grew with an average sheet length of 200–300 nm and graphitic edges composed of 5–6 graphene layers. In a similar process, Ostrikov et al. reported the vertical growth of graphene sheets using melted honeycomb as the precursor [60]. The growth of vertical graphene was carried out in the same conditions as above and showed an average length of 200 nm and a thickness of 2 µm for nanowall. Similar to the PECVD mechanism, these reports also state that the substrate temperature of the growth process is very low (400–450 °C) compared to thermal CVD.

From all the results mentioned above, CNWs possess a common morphology, even when synthesizing with different plasma techniques and at extremely different growth conditions. Thus, it is very important to understand the general growth mechanism of CNWs. In this review, an attempt is made to explain the general mechanism of CNW growth and the influence of different factors on growth and morphology. The upcoming sections are discussing the impact of various discharge parameters (gas species, plasma power, electric field, and substrate temperature) on the growth mechanism of CNWs.

## 3. Growth Mechanism of Vertically-Oriented Carbon Nanostructures in Plasma-Enhanced Chemical Vapor Deposition (PECVD)

**Figure 3:**
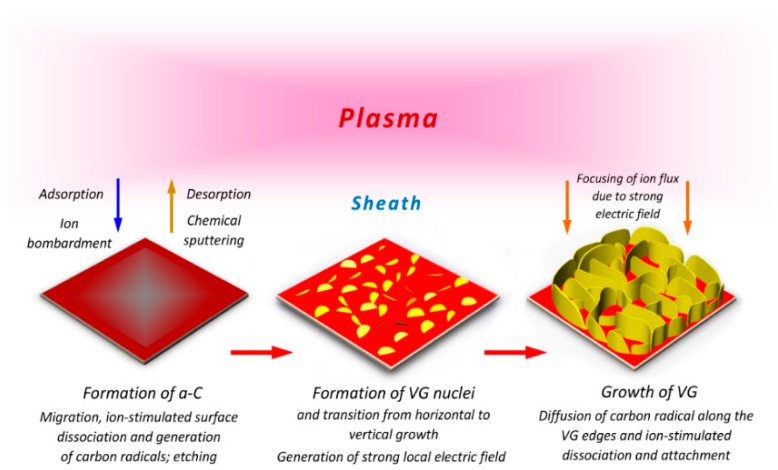
Schematic diagram of a plasma-enhanced deposition.

The common morphology of CNWs arises due to the common mechanism for the growth of CNWs, which can proceed through the production of radicals in plasma, plasma–surface interaction, nucleation, and coalescence of carbon radicals and area selective growth to the vertical orientation. A schematic diagram of the CNW growth mechanism is shown in Figure 3.

The ignition of plasma inside the chamber affects the initiation of CNW growth through the ion-stimulated plasma–substrate surface interaction and production of specific radicals for the growth through the dissociation of gas molecules. The substrate used for the deposition is in direct contact with the plasma and, thus, the ion stimulated plasma–surface interaction creates defects on the substrate surface, which acts as an immobilized free radical capable of forming dangling bonds with the radicals produced from the plasma. On the boundary between plasma and surface, a self-organized plasma sheath is formed, which modifies the energy and flux of the radicals to move towards the substrate to initiate the deposition. Formation of the nucleation site for vertical growth is the first step. The radicals dissociated from the gas source are absorbed or migrate to the surface and are attached to the defects via the dangling bonds. Absorption of this radical species in the early stage of growth forms a thin layer of carbon film with a thickness 20 nm parallel on the substrate called a buffer layer [61,62] (some authors report it as the carbidization layer [26] or graphitic layer [63]) composed of both graphitic and amorphous carbon. Along with the growth time, more carbon radicals get dissociated and attach to this layer in the form of carbon nanoislands, where nucleation initiates. These nanoislands or vertical graphene nuclei (VG nuclei) also have a dangling bond to attach more carbon atoms to it and act as the nucleation center for the growth of nanosheets. When a sufficient level of force is acquired at the grain boundaries of the sheets, it leads to curling the edges of top layer sheets vertically along with the direction of the electric field. The curling of the nanosheets to the vertical form is highly influenced by the plasma sheath, since the sheath contains a large number of incoming carbon species with high surface mobility and induces an electrical field perpendicular to the substrate. These vertically-oriented sheets grow faster compared to the parallel sheet, since the reactive carbon species are attached to the vertical graphene sheets more easily, and coalescence occurs. Thus, the parallel graphene sheets are shadowed by this faster vertical growth [64]. Eventually, the height of the graphene sheets increases with the growth rate rather than the thickness, resulting in the formation of nanowall. The growth of nanowalls increases with the growth time and they are grown throughout the substrate to form an interconnected structure with finite interlayer spacing [63]. The significant species responsible for the initiation, coalescence, and orientation has not yet been revealed well. However, radicals with high surface diffusion, such as carbon and small hydrocarbon/fluorocarbon (HC/FC) molecules, can provide edge growth of the CNWs.

Baranov et al. developed a theoretical model to investigate the key factors for the growth of vertically-oriented CNWs [65]. The model gives an insight into the processes taking place in the nucleation and orientation of CNWs in plasma systems. These calculations are fitted to a larger number of experimental data from various authors to match the macroscopic parameters to the microscopic growth process. This study proposes that ion bombardment and ion flux highly influence the growth of CNWs. Furthermore, the model agrees with the influence of surface activation via defect formation for the nucleation. The influence of the electric field on the vertical growth also confirms this and proposed that focusing ion current to the edges enhances the edge growth. This model also suggests that the sophisticated control over all these parameters will allow the formation of larger and denser arrays of CNWs. However, plasma surface interaction and surface activated defects can influence the growth mechanism up to the formation of the buffer layer [66]. Therefore, it is very important to understand the effect of various plasma-discharge parameters on the initial and vertical growth of CNWs after buffer layer formation. Apart from the plasma–surface interactions, the composition of plasma gases also determines the quality of prepared CNWs. In addition, there are other important parameters with which one can influence the CNWs’ growth and quality: Electric field determines the vertical orientation; growth time regulates the height of CNWs; temperature of substrate and gas pressure influence the morphology and growth rate; concentration of carbon radicals determines the rate of the deposition and morphology, etc. All the flexibility of plasma deposition offers a variety of CNWs with different properties. However, it can be a challenge to find optimum parameters for the optimal growth of CNWs for specific needs.

## 4. Influence of Gas Sources and Gas Proportion 

Considering the fact that CNWs are grown with a similar structure and morphology under various discharge conditions, reactive gas species play a key role in the growth of high-quality CNWs. The gaseous species for CNW growth can be classified as (i) carbon-containing precursor gases (HC or FC), which produce carbon radicals for the CNW growth via plasma-enhanced deposition; (ii) etchant gases for the removal of a-C (H_2_, O_2_), to assist with the growth of high-crystalline CNWs; and (iii) dopant gases (N_2_, NH_3_) when doping of CNWs is required. There is a variety of gas mixtures reported for the growth of CNWs, such as CH_4_/H_2_ [3,25], Ar/CH_4_/H_2_ [67], Ar/C_2_H_2_/H_2_ [68], Ar/C_2_H_2_/NH_3_ [69], CH_4_/NH_3_ [70], and C_2_H_2_/NH_3_ [71]. Many more are listed in Table 2, together with the significant effect of radical species on the CNW morphology.

The HC/FC gases and their mixtures with other gases (H_2_, O_2_, Ar, etc.) are the widely used carbon-containing gas sources for CNW growth, which dissociated in the plasma system to form various radicals, such as carbon dimers, small HC/FC radicals, and F/C radicals. Consider the HC precursor, which can effectively produce different hydrocarbon radicals as the provider of carbon dimer, by the plasma discharge. In the case of HC gases, for example, CH_4_ containing gas can easily produce CH_x_ radicals as the C_2_ provider for the CNW nucleation through the radical recombination and dissociation [74]. However, in C_2_H_2_ gas, carbon dimers were produced from the direct dissociation of HC≡CH with a strong C≡C bond [75]. The density of different radicals produced by different plasma sources may vary with the growth conditions, which further varies the morphological features of CNWs. In an RF plasma, a higher density of CH_x_ (x = 1, 2, 3) radicals mainly contributes to the production of carbon dimers. On the other hand, in an MW plasma, HC gas species are directly dissociated into CH_x_ and C_2_ radicals with approximately equal densities. Most of all, the experimental investigations confirm the importance of C_2_ radicals for the nucleation and coalescence of carbon nanosheets [75,76,77]. Investigations on the radical density of C_2_ radicals in MW and RFICP systems shows that the radical density of C_2_ ranging from 10^11^–10^13^ cm^−1^ is favorable for the initial growth of carbon nanostructures [78,79]. The growth of CNWs in an RFICP system by supplying CH_4_/H_2_ and C_2_H_2_/H_2_ gas mixtures was compared by Zhu et al. [53,77,78,79]. The thickness of the CNW synthesized using C_2_H_2_/H_2_, and CH_4_/H_2_ gas species was about 1–2 nm and less than 1 nm, respectively. The higher thickness in the first case is mainly due to the higher density of C_2_ radicals produced by the direct dissociation C_2_H_2_ gas. Teii et al. described an MW system that contains a large number of C_2_ radicals for synthesizing CNWs even in the hydrogen-poor condition by employing C_2_H_2_/Ar/N_2_ gas [75]. Similar to HC systems, FC gas mixtures are dissociating into a large number of CF_3_ radicals, which are providing the carbon dimers for the CNW growth. However, the absence of H atoms in the system slows down the growth initiation due to the inefficient abstraction of fluorine from CF_x_ radicals to produce C_2_ radicals. Therefore, an additional H source is coupled to the FC systems to achieve sufficient H radicals for the high-quality growth of CNWs. The mechanism of growth in the FC systems can briefly be described as: Production carbon atoms from fluorocarbon radicals, removal of F-atoms from carbon atoms by reacting with H radicals, migration or adsorption of C-atoms on the substrate surface, and formation of nanoislands on the surface through dangling bonds. Finally, the nanostructure is continuously grown on the surface [49].

**Figure 4:**
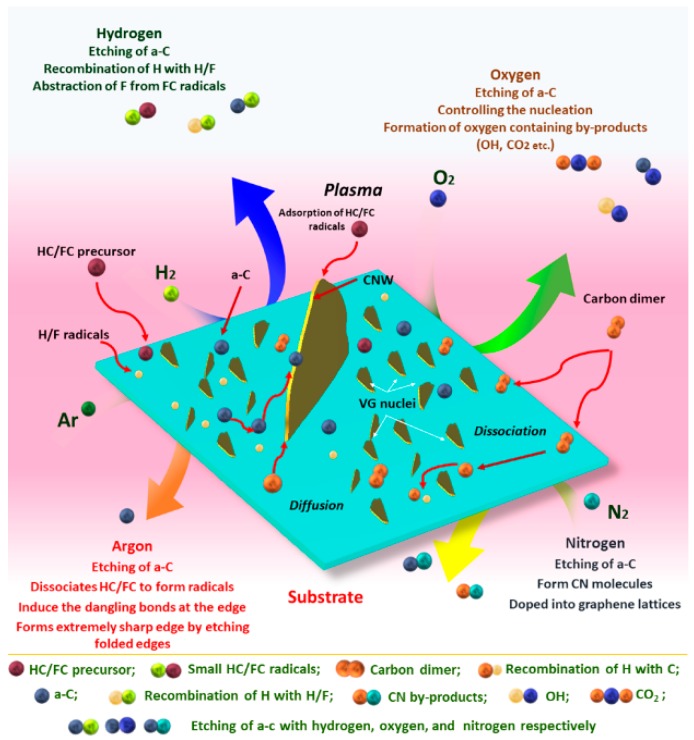
Effect of different radical species on the growth of carbon nanowalls.

Following the precursor gases, etchant gases are mainly supplied to the system for the effective removal of the amorphous interface phase and to enhance the growth. As mentioned in the above section, the assistance of H for the abstraction of bonding atoms from the radicals on the surface is explained in various reports, mostly in the FC gas plasma [41,49,80,81]. The presence of H atoms in the plasma system assists in the nucleation of the carbon radical and simultaneously acts as the etchant gas for a-C [3,53,82]. Furthermore, H atoms are more reactive with a-C atoms than the *sp*^2^ and *sp*^3^ hybridized carbon atoms, which helps with the removal of the a-C phase and results in the formation of highly crystalline CNWs with sharp edges. Similarly, some reports show that oxygen and nitrogen also assist in the effective removal of a-C from the surface. Kondo et al. explained the effect of O_2_ radicals on the successful growth and removal of a-C on the substrate [49]. The study investigated the nucleation and growth of CNWs using FC gases with and without the presence of oxygen. The presence of O radicals is capable of reducing the defects and suppressing the carbon nucleation and retarding the formation of the interface layer, which leads to the formation of carbon nanostructures without any interface layers. Additionally, O_2_ promotes the vertical growth of CNWs from the nuclei by removing this horizontal interface nucleus. Hydrogen–oxygen combined OH radicals are also capable of removing the a-C carbon. The reports by Bo et al. [56] and Chateai et al. [83] indicate that OH radicals have much more capability than H atoms for the effective removal of a-C. The advantage of nitrogen as an etching agent was described by Chuang et al. by supplying C_2_H_2_/NH_3_ as the gas source. Here, the NH_3_ molecules dissociate into different radicals and provide sufficient H atoms for the efficient removal of a-C [73]. Neutral gases, like argon, are also supplied to the plasma system for enhanced CNW growth. Most of the MW-based growth by HC gases is assisted by the argon, since Ar has a higher excitation and ionization potential compared to hydrogen. Thus, the interaction between the plasma activated species and Ar is dominated by the elastic collisions and the energy loss through the inelastic collisions reduced. In this way, the addition of argon increases the electron temperature and enhances the plasma stability. Moreover, the addition of argon into the plasma system promotes the production of C_2_ through the direct dissociation of gas sources. There are several reports observing that higher Ar concentration in the plasma enhances the carbon dimer concentration, which is a benefit in the high degree of graphitization [75]. The presence of Ar enables to provide the high-quality growth of CNWs by increasing plasma stability and moderating the growth rate. The main features of the influence of different gaseous species on the growth of CNWs are shown in Figure 4. Along with the source and etchant gases, some dopant gases are also used when CNWs requires doping. Doping of CNWs and surface functionalization of CNWs by various gases for tailoring the properties are discussed in detail in this review later. In addition to the mixture of source gases, the concentration of the gas species, the flow rate of gases, and the proportion of the gas, mixtures also significantly affect the nucleation and coalescence of CNWs.

**Figure 5:**
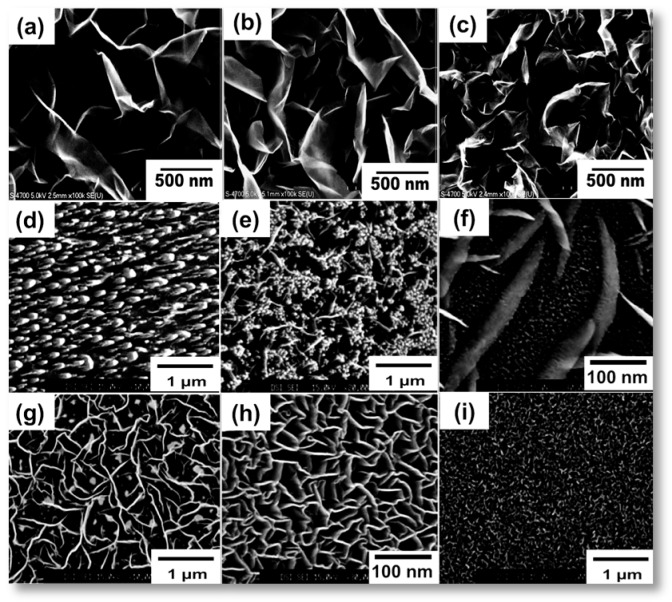
SEM images of graphene nanowalls (GNWs) with different CH_4_ concentration (**a**) 10%, (**b**) 40%, (**c**) 100%. Reprinted with permission from the authors of [84]. Copyright Elsevier 2004. SEM images of carbon grown at different H_2_/CH_4_ flow rate ratios: (**d**) 30, (**e**) 15, (**f**) 10, (**g**) 6, (**h**) 4, (**i**) 1 sccm. Reproduced with permission from [85]. Copyright Royal Society of Chemistry 2004.

The proportion and flow rate of gas into the plasma system also has a significant role in the growth of CNWs. The requirement of optimum concentration of carbon and the etchant gases for the growth mechanism was already discussed in the above section, which highly determines the structure, morphology, and properties of the CNWs deposited on the substrate. The experimental study on the influence of the concentration of carbon gases on the growth of CNWs was carried out by Wang et al. [84] The concentration of precursor CH_4_/H_2_ gas was controlled by various flow rates (0–100%). The study reveals that the density of GNW sheets was highly influenced by the CH_4_ concentration, which is responsible for the nucleation of carbon radical and initial growth. A higher nucleation rate with small lateral size was observed at a higher concentration of precursor gas (40–100%). The various structures of CNWs formed at the different CH_4_ concentration are shown in Figure 5a–c. Wu et al. gave a good description of the effect of flow rate of the gaseous mixture on the growth mechanism [85]. Figure 5d–i shows the SEM images of different CNW morphologies according to different flow rates in his experiment. The CNWs were grown on Au (cc. 20 nm) coated Si substrates using CH_4_/H_2_ as a precursor gas in an MWPECVD system at growth temperature of 650–700 °C. The impact of flow rate on the structure and morphology of CNW growth is observed by the varying flow rate of the CH_4_/H_2_ mixture from 30, 10, 6, 4, to 1 sccm. At 30 sccm, a-C with column structures was observed. A further decrease in the flow rate led to the evolution of tube-like carbon structures. Finally, the high-quality CNWs were formed at a flow rate between 4–8 sccm. Moreover, the further decrease in the flow rate led to the formation of a-C on the substrate. This indicates that the morphology of the final product, even in the same system with the same discharge conditions, was highly affected by the flow rate of gas into the system.

**Figure 6:**
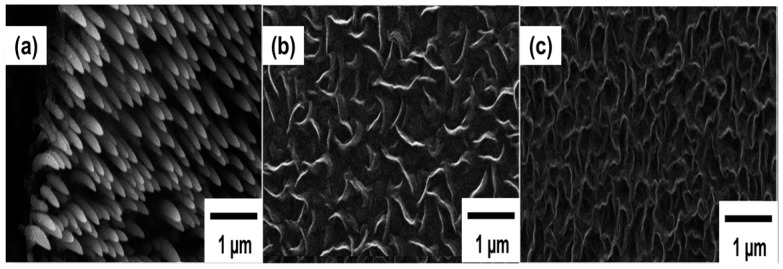
SEM images of (**a**) a tilted view of carbon nanofibers (CNF) and a top view of (**b**) freestanding CNW and (**c**) interconnected CNW. Reproduced with permission from [57]. Copyright Royal Society of Chemistry 2016.

Lehmann et al. described the effect of the flow rate of the aromatic precursor on the growth of CNWs [57]. Aromatic paraxylene (P-xylene) was inserted to an RFICP system as the precursor. Plasma power of 150 W, a substrate temperature of 450 °C and pressure between 4.6–7.5 Pa for 20 min were the observed optimum conditions for the CNW growth. The flow rate of the liquid precursor to the system varied from 0.5–5 mL/h (0.01–0.08 sccm) to obtain different morphologies. SEM analysis on the deposited material is shown in Figure 6a–c, which displays the growth of carbon nanofibers (CNF) with a height of 2.2–6 µm, which were produced by a flow rate between 3–5 mL/h. Free-standing CNWs with a height of 1.4–2.2 µm grew at a flow rate of 1–2 mL/h. Interconnected CNWs with a height of 1.1–1.6 µm were deposited at a flow rate of less than 1 mL/h. The investigation stated that the decrease in flow rate increases the interconnection between the nanostructures to form interconnected CNWs from CNF and the height decreases in the order of 33%.

Similar to these carbon-containing gases, the flow rate of etchant gases also influences the growth mechanism. Kondo et al. investigated the effect of the flow rate of H_2_ gas on the nucleation and growth of CNWs [86]. A multibeam RFICP system was used for the investigation by supplying an FC precursor source and H_2_ as the etchant gas. The H_2_ gas was inserted into the system by the direct radical injection mechanism and by an radical ICP system. The morphological changes of the CNWs by the influence of H radicals were studied by the synthesis of CNWs for 35 min at various flow rates of 0, 3, 5, 7, and 10 sccm. A thin layer of carbon was formed on the substrate without an H radical. An increasing H radical flow rate up to 3 sccm initiated the carbon nanoparticle deposition. A further increase to 5 and 7 sccm produced CNWs with an interlayer spacing of 10–20 nm and sheet thickness of less than 5 nm. The height of CNWs was also influenced by the H radical flow. The maximum height for CNWs was observed at a flow rate of 5 sccm. In all other cases, the height decreased with increasing the flow rate, that is, the optimum density of the H radical inside the system highly influenced the vertical growth of CNWs. Suzuki et al. have also investigated the effect of hydrogen on CNW growth by the MW system [87]. Compared to the previous case, Suzuki et al. investigated the variation in the morphology by changing the H_2_/CH_4_ ratio in between 0–4. The morphology of CNWs varied from a wavy and densely distributed to a linear and sparsely distributed manner on the substrate with increasing the flow rate ratio. Moreover, the wall height increased with increasing the H_2_/CH_4_ flow ratio. At lower flow rate ratios, the hydrogen did not actively participate in the graphitisation due to the poor H radical condition, while at a higher flow rate ratio, hydrogen participated in the effective removal of a-C from the substrate to form high-quality CNWs. The enhancement of the morphology and crystallinity of CNWs by the addition of O_2_ was described by Takeuchi et al. [88]. CNWs were synthesized by RFICP with a radical injection system by employing a C_2_F_6_/H_2_ mixture. The addition of N_2_ and O_2_ to a system slightly changed the morphology and properties of the CNWs. N-doped CNWs were formed by the insertion of N_2_ with higher crystallinity and density. Thus the proportion and optimum flow of various gas species into the system influence the overall growth mechanism of CNWs, which in turn altered the pressure inside the plasma system. 

The growth rate of CNWs varies with varying flow rates, which also changes the pressure inside the system, a logical consequence. Thus, the growth of CNWs can also be influenced by the operating pressure. In low-pressure RFICP systems, the total pressure inside the chamber is controlled by regulating the flow rate of gases inserted. The inductive coil stimulates the magnetic field and sustains a breakdown voltage for the dissociation. Therefore, the influence of pressure on the growth can be explained as similar to the effect of the flow rate of gases. On the other hand, in RFCCP, DC, and MW coupled with electrode based plasma systems, dissociation of gaseous species takes place under a certain breakdown voltage, which is described as the function of pressure (*p*) and the distance between the electrodes (*d*). This influence of breakdown voltage on gas dissociation is described by Paschen’s law [89]. A Paschen’s curve indicates that the breakdown voltage decreases with decreasing *p* and *d*. Most of the PECVD synthesis is carried out at low pressure. Thus, the *p-d* values should always be less than its critical values. In most of all the PECVD systems, the distance between the electrode is usually fixed as a constant. Therefore, the breakdown voltage and growth can be influenced by varying the pressure, which means the value of *p* has to be lower than its critical value. The change in pressure also determines the mean free path of electrons, since the electron mean free path is inversely proportional to the pressure. The low pressure inside the system can increase the mean free path of electrons, which increases the (i) electron temperature via two successive collisions and (ii) stability of plasma. Following this, the plasma becomes capable of providing a sufficient amount of electrons with a higher energy and enhances the ionization rate, which results in the optimal growth rate of CNWs. Low pressure means low flow of gas species through the system and lower growth rate. However, the quality of CNWs at low pressure could be higher than the high-pressure plasma, since the lower growth rate effectively removes the a-C from the surfaces. Hiramatsu et al. reported the influence of pressure for the synthesis of vertical nanographene networks to determine the balance between the gas composition [90]. A mixture of CH_4_/Ar inside an RFICP was used for the growth of CNWs at total pressure ranges from 15 to 20 mTorr. This low-pressure operation in an ICP system was capable of enhancing the effective ion bombardment with the surface for the successful nucleation of CNWs. However, the wall density decreased and interlayer spacing increased with the slight increase of total pressure. The investigation by Takeuchi et al. showed that the pressure inside the system influences the growth rate and the radical density, as well [91]. A very high frequency (VHF) CCP+MW PECVD system employed with a C_2_F_6_/H_2_ gas mixture was used to study the effect of pressure on the radical density. The increase in pressure from 13.3 to 80 Pa kept the C atom density constant, while the H atom density increased. The important finding in this study was that the ratio between H/CF_x_ in an FC system is important for the formation of CNWs. The height of the deposited CNWs decreased, and interlayer spacing increased with the increase in pressure. The graphitization of well-oriented CNWs increased with changing pressure to the maximum as well. Similar to the low-pressure PECVD systems, the growth of CNWs at atmospheric pressure (atm) also illustrated the effect of pressure on growth. Bo et al. reported the high growth rate of CNWs using atmospheric normal pin-to-plate DC glow discharge plasma [56]. The strong electrical field produced near the tip enhanced the initiation and growth of CNWs. The gas flow rate was elevated to reach high pressure, which further improved the growth rate. Yu et al. also reported the advantage of pin-to-plate DC plasma to produce high electric field near the tip with atmosphere conditions for the enhanced growth [14]. These all studies confirm that the mixture of gas species, the proportion of gas mixtures, and flow rate of gases play a significant role in the growth mechanism. Ostrikov et al. explained that the formation of nanostructures on the substrate in a PECVD process was mainly influenced by the insertion and consumption of gas species, where the consumption of gas species was regulated by the substrate temperature, which in turn was affected by plasma power [92]. In this aspect, it is very important to find the influence of plasma power, electric field, and substrate temperature on the synthesis of CNWs.

## 5. Electric Fields and Plasma Power 

**Figure 7:**
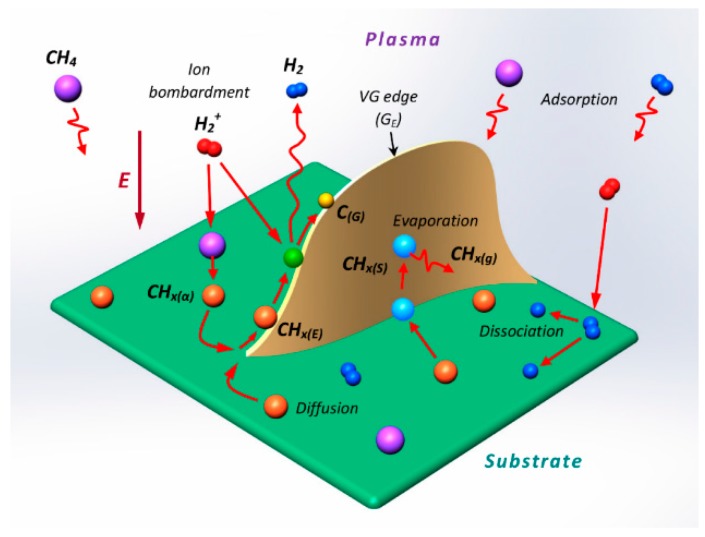
A schematic explanation of the CNW growth model. E: The direction of an electric field; CHx_(g)_: HC growth species; C_(G)_: Graphene sheets; H: Atomic hydrogen used as an etchant. CH_x(α)_: a-C etched along with H atoms in the form of hydrocarbon (HC); VG edge: Edges of vertically-oriented CNWs. Reproduced with permission from [63]. Copyright Elsevier 2007.

Nanowall growth is initiated by the nucleation and coalescence of carbon radicals to form graphene sheets on the substrate. Thus, the curling of these sheets to vertical orientation is the following step. As described in the chapter about the growth mechanism, the curling of nanosheets into a vertical orientation is highly influenced by the electric field. Yang et al. explained that the variation in the microstructure during CNW deposition was mainly due to the high electrical field perpendicularly aligned to the substrate, which is provided by plasma [93]. This electrical field promotes the generation of *sp*^3^-hybridized C atoms as the nucleation center for vertical growth. Wu et al. explained the effect of the strong lateral electrical field on the CNW growth in an MW system [85]. The study revealed the influence of the electric field with surface plasmons induced by gold particles to alter the growth of nanowalls in very localized areas. A growth model by Zhu et al. proposed that the electric field in a plasma system enhances the growth through edges and induces the orientation perpendicular to the substrate [63]. A schematic of the growth model using CH_4_/H_2_ gas species for the growth of CNWs is presented in Figure 7. The curling of nucleated graphene sheets was initiated when the *sp*^2^ bonded network in the graphene sheets overcame the activation energy barrier for the distortion. A strong electric field perpendicular to the substrate helps to overcome the activation barrier on the edges and sheets bend into the direction of the electric field. The elimination of dangling bonds at the edges by the etchant gases reduced the total energy, which further reduced the probability of edges to bond with each other. A theoretical model on the key factor for the growth of CNWs by Baranov et al. explained that an increased electric field helps to focus ion current to the sharp nanoflake edges on enhancing the height of nanowall [65].

**Figure 8:**
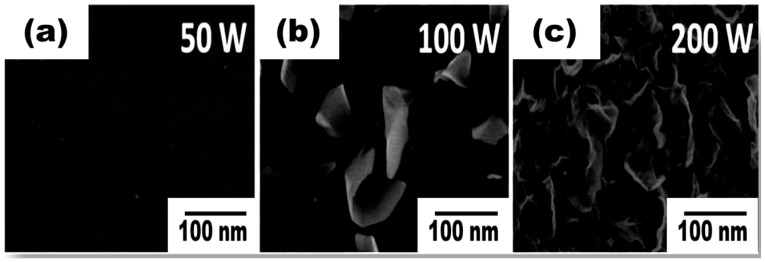
SEM images of GNWs under different plasma power, (**a**) 50 W, (**b**) 100 W, (**c**) 200 W. Reproduced with permission from [93]. Copyright Royal Society of Chemistry 2013.

Similar to the radical species, neutral gas species also play an important role in the growth mechanism. The collision of neutral atoms and ions, which accelerate through the plasma sheath, enhances the growth rate and creates more defects on the nanowalls [63]. The intensity of the electric field can be externally controlled by varying the plasma power. Zhu et al. described that the growth rate and morphology of CNWs were increased with increasing the power from 500 to 1200 W [63]. High plasma power induces a high electric field on the plasma sheath above the substrate, causing elevation of the substrate temperature and forcing the nanosheets to curl up to a vertical form with a higher growth rate. Yang et al. also explained the better growth of CNWs at a higher power by regulating RF plasma power from 50 to 200 W [93]. The SEM images of CNWs deposited by different plasma power are shown in Figure 8, where enhanced growth is observed at 200 W. Therefore, the formation of the carbon nanostructure using a high plasma power shows a strong correlation with the electric field effect to form a vertical structure.

## 6. Substrate Temperature

**Figure 9:**
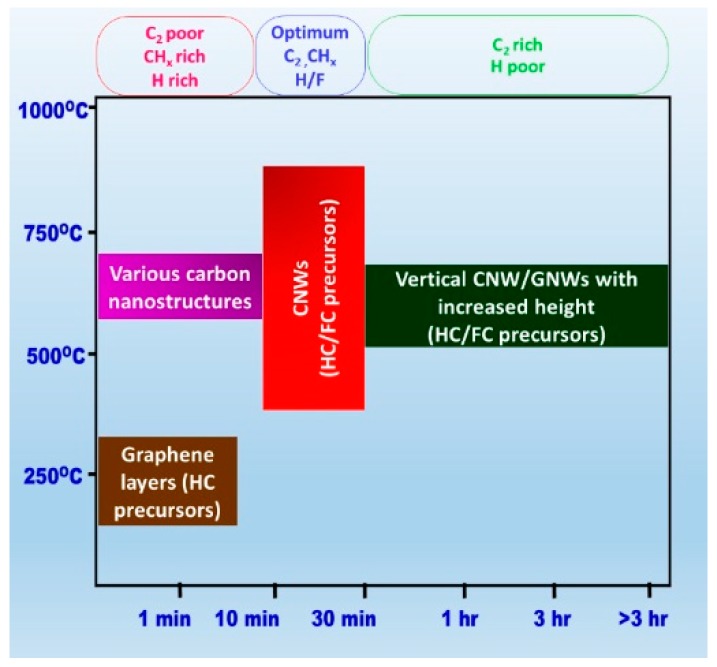
Summary of time–temperature growth regimes for the initial growth of different carbon nanostructures.

The energy transfer into a PECVD system is carried out mainly by the transfer of electrical energy via plasma, and thermal energy via heating effects. Compared to the thermal CVD systems, growth in PECVD occurs at a relatively lower temperature due to the effects of plasma activated species. In low-pressure PECVD systems, the electrons gain more energy than the ions in plasma through the external electric field. The elastic collisions of electrons can transfer the kinetic energy in relatively smaller amounts than the ions. The electron temperature is much higher than the temperature of ions, but the transfer of energy to other plasma components from electrons is lower because of the extremely low mass of electrons [94]. Thus, the heat transfer inside the plasma becomes low (<1 eV). However, the higher temperature generated by the strong electric field with high-intensity plasma can offer the possibility to transfer the heat to the substrate more effectively by reactive species and ion-based reactions on the surface [95,96]. In addition to this, the neutral gas temperature in high-density plasmas is typically raised, too. This helps the active species to enhance the nucleation and coalescence to form CNWs by forming dangling bonds to the surface defects. The studies on the synthesis of carbon nanostructures show that if the temperature is not fixed exactly, the plasma heating effects promote the substrate temperature and growth. Therefore, the approximate growth–temperature regimes for various carbon nanostructures can be expected as: Graphene growth 400–700 °C [97], initial growth of CNWs 400–600 °C [64,98] and vertical growth of CNW/GNW 500–850 °C [41,98]. Figure 9 shows the growth regimes to initiate the growth to form different carbon nanostructures.

**Figure 10:**
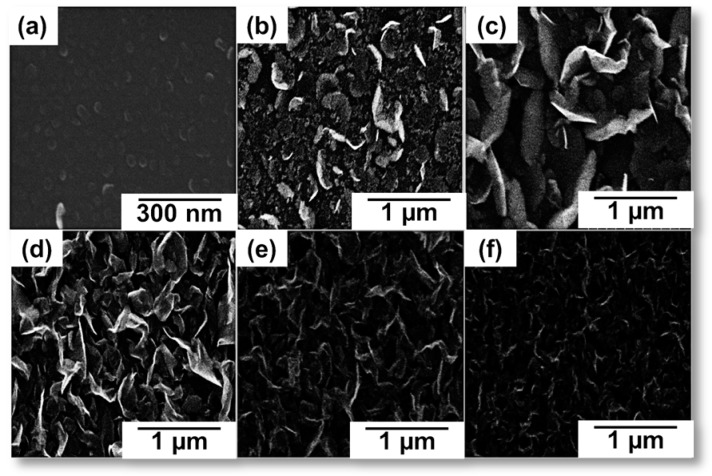
SEM images of the CNWs according to the following growth temperatures: (**a**) 700 °C, (**b**) 750 °C, (**c**) 800 °C, (**d**) 850 °C, (**e**) 900 °C, and (**f**) 950 °C. Reproduced with permission from [98]. Copyright Elsevier 2014.

Kim et al. studied the effect of substrate temperature on CNW growth using the CH_4_/H_2_ gas mixture in an MW system [98]. The CH_4_/H_2_ gas mixture was introduced into the chamber at a flow rate of 30/15 sccm and the pressure inside the chamber was maintained at 10^−2^ Torr. The CNWs were synthesized on the *p*-type Si substrate at a plasma power of 1000 W. The surface images of CNWs at different substrate temperatures are presented in Figure 10a–f. The deposition of carbon is observed from the temperature of 750 °C. Elevation of temperature to 800 °C destroyed the nucleated structure of carbon atoms and flattened on the substrate. On further increase to 850 °C, 900 °C, and 950 °C, the vertical growth of highly interconnected CNWs was observed with the reduced surface area. The elevation of the temperature also resulted in changes in the surface properties. The contact angle of the CNWs increased linearly until 850 °C and then decreased, where the highly interconnected CNWs with the low surface area was formed. Thus, the elevation of temperature can enhance the growth rate of CNWs and modify the morphology, and further increase leads to the modification of the surface properties.

Once all the discharge parameters are set for the growth of CNWs and plasma generation, the growth of CNWs is initiated by the substrate surface activation, which promotes the selective area growth of CNWs. The growth mechanism and properties of CNWs can be influenced by the plasma parameters. On the other hand, the properties of CNWs can be modified by different plasma treatments after the growth, which is one of the main advantages of plasma-assisted techniques. The following part of the review will mainly focus on the surface treatment of CNWs with plasma generated by different gases.

## 7. Plasma Assisted Surface Modification and Doping

Enhancing the properties of CNWs specifically through surface treatment or surface functionalization is the main advantage of plasma techniques. Functionalization of the surface is a simple tool for incorporating different functional entities, such as reactive radicals, metallic nanoparticles, and pendant molecules, into the graphene lattice and modifying their properties for various applications. Plasma functionalization can be performed in two ways: (i) In-situ treatment, modification, and functionalization of CNWs during growth, when dopant gas is added into the carbon precursor mixture; or (ii) through post processing treatment. Effects of the in-situ treatment with different gases during growth were already discussed in the *Influence of Gas Sources and Gas Proportion* section of this article. Thus, this section is mainly focused on the plasma post processing on CNWs by using different plasma gas compositions. Usually, a hydrogen plasma treatment helps to produce high-quality carbon nanostructures by removing the HC groups and contaminants and is also observed through changed wettability of the surface [19,99,100,101]. Adding oxygen to plasma synthesis of CNWs results in the etching of a-C and the formation of oxide groups and carbonyl groups [19,49]. However, high energy plasma with a high density of oxygen ions can destroy the crystallinity of carbon nanostructures through continuous etching [102]. Oxygen plasma treatment is also an effective tool for tuning the band gap of graphene sheets, since it disrupts the atomic ordering in the graphene layer and forms an oxygen-containing functional groups [103]. The use of nitrogen plasma post-treatment enhances the doping and grafting of the amino functional groups into the CNW structure, which modifies the electrical, electronic, and field emission properties, which can be beneficial also for improving the sensing capability of the material [75,104,105]. Similarly, argon plasma post-treatment influences both electronic and surface properties of carbon nanostructures [17,106]. Argon plasma treatment is generally used to enhance the removal of a-C, mostly through kinetic interactions, and by the prolonged Ar treatment on the edges of nanowalls results in the thinning of the wall and enhances the electronic properties and field emission characteristics [17]. Use of fluorinated gases in post-treatment can lead to controlling the surface wettability properties of CNWs [107]. Such effects are also seen when adding fluoro-based gases into in-situ synthesis systems. Adding boron to plasma gas mixtures during synthesis leads to the formation of B-doped CNWs, which enhances the field emission properties and also participates in the bandgap tuning [108,109]. Additionally, the electrochemical properties of CNWs were enhanced by boron doping [110]. The optical and electronic properties of carbon nanostructures can be tuned by using chlorine-containing plasma, too [111]. To summarize, the use of plasma treatment can lead to either the formation of functional groups (e.g., carbonyl, hydroxyl, carboxyl), an increase in the number of defects due to the removal C atoms from the lattice (e.g., by Ar treatment), or doping of impurities into the graphene lattice (e.g., N and B-doping). Various plasma-assisted surface treatment/doping techniques using different gases reported so far are summarized in Table 3.

Vizireanu et al. investigated the effect of post surface plasma treatment of CNWs using H_2_, O_2_, N_2_, tetrafluroethane (C_2_H_2_F_4_), and sulphur hexafluoride (SF_6_) plasmas [19]. Randomly distributed CNWs with an average height of 1.5 µm, a length of 0.8 µm, and a thickness of 10–20 nm were used in the experiment. Morphological investigation unveiled that the post-treatment using H_2_, O_2_, and N_2_ caused thinning of the walls and branching towards each other. C_2_H_2_F_4_ plasma treatment caused the formation of a fluorine thin film on the CNW surface, while SF_6_ plasma treatment led to the rounding of edges by smoothening the sharp edges of CNWs. The formation of carbonyl, aldehyde, epoxy, peroxide, and aromatic quinoides during plasma post-treatment by all the gas mixtures was observed through vibrational spectroscopic analysis, and the formation of additional FC groups in the C_2_H_2_F_4_ and SF_6_ plasma conditions was also described. The wettability studies on the materials described the increased hydrophilicity of the CNWs after H_2_, O_2_, and N_2_ plasma treatments, while the formation of fluorocarbon groups on the structure by C_2_H_2_F_4_ and SF_6_ plasma treatment resulted in the superhydrophobicity of CNWs. In most hydrogen-containing plasma treatments, hydrogen is used as the in-situ agent to control growth and purity, due to the critical role of hydrogen for growth discussed earlier. However, hydrogen plasma post-treatment is mainly used for controlling the surface wettability by forming H-bonds on the edges and leads to the formation of superhydrophilic surfaces, which are widely used for sensing applications. Jiang et al. found that the hydrogen–plasma post-treatment can remove the oxygen atoms absorbed in the nanowall surface, which enhances CNWs adsorption capabilities [101]. Hydrogen plasma post-treatment is capable of modifying the surface through strong etching and forms a roughened layer on the nanowall surface with a large number of graphite bumps. Elias et al. showed that hydrogenation in graphene lattices could change the hybridization of *sp*^2^ bonded carbon atoms to *sp*^3^ bonds, which further results in the transformation of band structure from semimetal to insulators [133]. The term for this process is bandgap opening, which is an important aspect for electronic device applications.

**Figure 11:**
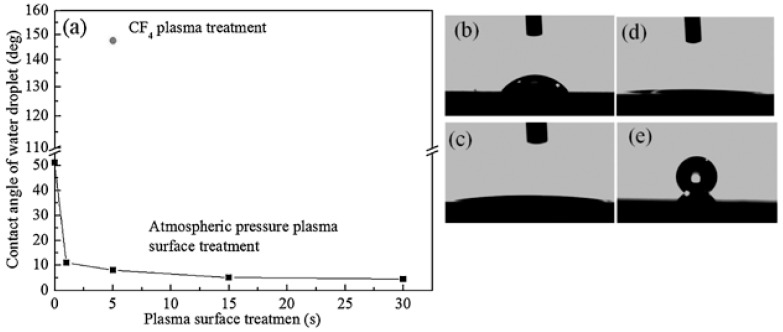
(**a**) Contact angles of water droplets on CNWs as a function of the plasma treatment time, (**b**) as-grown CNWs, (**c**) CNWs after Ar atmospheric pressure plasma treatment for 5 s, (**d**) CNWs after Ar atmospheric pressure of plasma treatment for 30 s, and (**e**) CNWs after CF4 plasma treatment for 5 s. Reproduced with permission from [106]. Copyright John Wiley and Sons 2013.

In contrast to hydrogen plasma treatment, fluorine-containing plasma post-treatment resulted in the hydrophobic nature of the surface (superhydrophobic in some cases) by forming F-terminated bonds on CNWs. This was also demonstrated in the study by Vizireanu et al. mentioned above. Furthermore, Watanabe et al. also came to the same conclusion that H-terminated or O-terminated CNWs have a hydrophilic nature, whereas F-terminated CNWs have a hydrophobic nature. The as-synthesized CNWs were treated with Ar plasma at atmospheric conditions, which leads to the formation of H/O-terminated CNWs by the incorporation of oxygen into the lattice through the dissociation of oxygen molecules in the air, while CF_4_ plasma led to the formation of F-terminated CNWs [106,134]. Figure 11a shows the water contact angle (WCA) of CNWs as a function of plasma post-treatment time. It is observed that Ar plasma treatment makes the surface more superhydrophilic through surface oxidation during the treatment. On the other hand, treatment with CF_4_ plasma makes the surface more superhydrophobic. Deposition of a fluorinated polymer layer by the CF_4_ radicals causes a superhydrophobic effect on CNWs. Thus, the formation of O-terminated CNWs leads to hydrophilic surfaces, whereas that of F-terminated CNWs by FC plasma leads to hydrophobic CNW surfaces. The effect of Ar post-treatment on the enhancement of field emission properties of CNWs was explained by Qi et al. [125] and Cui et al. [135]. The Ar post-treatment was efficiently removing the a-Cs, sharpening the edges, and removing the folded edges. The atomically thin edges of CNWs acquired by the optimum Ar plasma post-treatment show the maximum field emission properties. Ar ion bombardment on to the CNW edges causes the thinning of edges and created several defects on the edges, and these defects act as the additional emission sites, resulting in the enhanced field emission.

An oxygen plasma post-treatment influences both the edges and the interstitial sites of the CNWs to form different functional groups. The presence of the O-terminated carbon structure improves the surface wettability properties, which also increases the reaction with electrolytes in energy storage devices. Sahoo et al. reported the effect of oxygen plasma treatment to enhance the electrochemical performance of CNWs [136,137]. Formation of different oxygen-containing functional groups after oxygen plasma post-treatment enhances the capability of CNWs to interact with electrolyte rapidly, which further increases the specific capacitance of the material. Chen et al. also reported the effect of oxygen plasma post-treatment in enhancing the reaction of CNWs with enzymes for electrochemical sensors to detect lactate enzyme [138]. Gokus et al. and Wang et al. explained the effect of oxygen plasma treatment on graphene to modify the photoluminescent (PL) properties [120,139]. The significant amount of oxygen in the graphene lattice enhances the PL properties of CNWs by the transition of energy from π band to the defect energy band related to oxygen atoms. 

**Figure 12:**
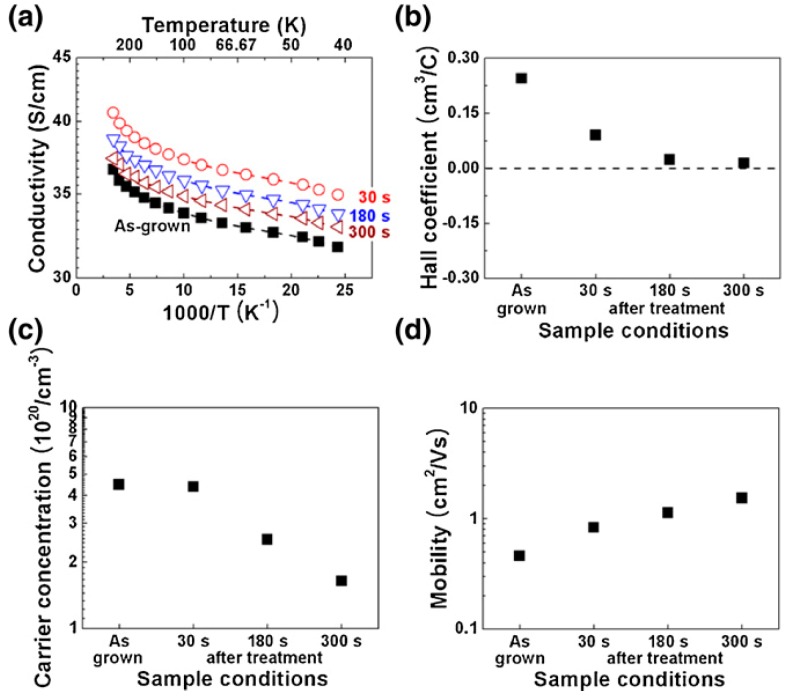
Temperature dependences of (**a**) electrical conductivities, (**b**) Hall coefficients, (**c**) carrier densities, and (**d**) carrier mobilities of the CNWs before and after post-growth N_2_ gas plasma treatments. Reproduced with permission from [130]. Copyright The Japan Society of Applied Physics 2014.

Nitrogen plasma post-treatment is widely used for incorporating nitrogen to the graphene lattice to produce N-doped CNWs. The nitrogen plasma post-treatment can significantly enhance the electrical and electronic properties of the CNWs. Cho et al. modified the conductivity and carrier mobility of CNWs by nitrogen doping while keeping the morphology and edges of CNWs intact [130]. Compared to in-situ N_2_ plasma during the growth of CNWs, N_2_ post-treatment on CNWs shows an improved carrier concentration and carrier mobility. The former leads to bulk nitrogen doping and consequential formation of higher topological defect densities and a decrease in the conductivity; the latter largely influences only the edges of CNWs, where the N atom is attached to the graphene edges and results in the higher charge carrier mobility and, thus, conductivity. Figure 12 shows the improved electrical properties of CNWs after nitrogen plasma post-treatment.

**Figure 13:**
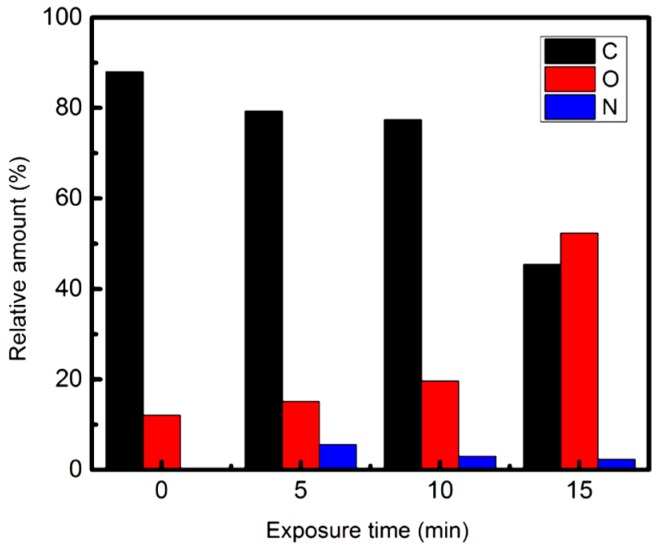
Evolution of the elemental composition of N-graphene with plasma treatment time (*P* = 600 W, N_2_–Ar (10–90%), *p* = 1 mbar). Reproduced with permission from [104]. Copyright IOP publishers 2016.

The concentration of bulk N-doping during growth can be controlled by the flow rate of nitrogen to the system, which was shown by Takeuchi et al. in the FC/H_2_ plasma synthesis [126]. The inclusion of N in the lattice changes the conduction type of CNWs to *n*-type from *p*-type. Uniform distribution of N-doping was controlled by the flow rate of N_2_ gas, where N-concentration increased with the increase in N_2_ flow. Dias et al. investigated the amount of nitrogen doped into CNW lattice by nitrogen plasma post-treatment and reported a high doping level of 5.6% [104]. The Ar–N_2_ plasma post-treatment was used for nitrogen doping on the self-standing graphene sheets. The evolution of the concentration of nitrogen on the graphene lattice with respect to plasma treatment time is displayed in Figure 13. The nitrogen plasma treatment leads to the formation of different N-graphene by forming pyrrolic-N, pyridinic-N, and quaternary nitrogen. Recently, Bundaleska et al. reported the large-scale synthesis of 0.4% N-doped free-standing graphene sheets via a highly cost-efficient microwave plasma system [58]. The in-situ N-doping was carried out using ethanol and ammonia as the precursors, with IR and UV irradiation post-treatment. The irradiation causes heating, which activates the defects through subsequent bond recombination and enhances the capability of N-doping. Tatarova et al. explained the synthesis of free-standing N-doped graphene via MW plasma system and reported 0.2% of N-doping [140]. The electrochemical properties of N-doped graphene showed a good electrochemical response, with negligible interfacial resistance. Chi et al. reported the higher concentration of N-doping on CNWs at 7.6%, which is suitable for use as an electrode for electric double layer super capacitors [141]. Yen et al. showed that the in-situ doping of N in CNWs exhibits maximum specific capacitance in aqueous electrolyte [142]. Wang et al. reported the photoluminescence effect from the N-doped carbon tips [143]. Bundaleska et al. reported the highest level of N-doping up to 25% on graphene sheets through Ar–N_2_ post-treatment in an MW plasma [144]. The highest level of N-doping into graphene lattice can be suitable for various applications. All the investigations on N-doped CNWs confirmed the enhanced electrical properties of CNWs through nitrogen doping.

The complex behavior of plasma surface chemistry on the growth and modification of CNWs is not fully understood. However, functionalization is mainly occurring at the edges, which helps to control the surface chemistry of CNWs. Attaining substantial edge growth rather than the production of a large number of nucleation centres is one of the main future goals through different plasma treatments. Most recently reported CNWs contain an amorphous structure in their morphology, which can be reduced by in-situ treatment with the optimum concentration of etchant gases to achieve high crystalline CNWs with high quality. Plasma surface treatment always opens the chances for surface functionalization and doping on the CNW structures. The functionalization on the graphitic edges of CNWs through plasma post-treatment using different gases may result in the development of a new electronics era based on edge electronics. Tuning electrical, optical, morphological, and chemical properties of CNWs using plasma surface treatment can be applied in the fields of electronics, optics, energy, and so on, which are yet to be explored in depth.

## 8. Challenges and opportunities

The synthesis of open boundary structured CNWs has been widely investigated. The main challenges of CNW synthesis are structure controlled growth, large-scale production, high growth rate, and low-temperature growth. The vertically-oriented structure and morphology with thin edges of CNWs strongly enhance their surface and electrical properties, which are widely applicable to potential applications. Therefore, a better understanding of the optimum conditions for the structure controlled growth is one of the main challenges to be resolved, which can be explained by studying the influence of discharge parameters on the growth of CNWs. The challenges arise due to different growth parameters such as: Low-pressure plasma results in a low growth rate, high temperature, and high power plasma-treatment, sometimes destroying the structure of CNWs and the presence of dominant amorphous nature in hydrogen-poor conditions. Thus, it is critical to choose the suitable gas source with an optimum flow rate and proportion to maintain the optimum pressure inside the system for the enhanced growth. Additionally, the selection of a-C etchant source gases is critical to increasing the crystallinity of CNW. The growth rate under different plasma conditions reported until now is not sufficient for industrial-scale production. Until now, the mostly reported plasma-enhanced growth of CNWs was carried out via the gas phase deposition of carbon radicals onto the substrate. There are very few works available on the growth of CNWs or vertical carbon nanostructures via a surface growth mechanism or using aromatic precursors. The surface growth of vertically-oriented structures may give more control over the thickness and size of the nanostructures. Furthermore, it is critical to fabricating isolated free-standing CNWs, which can be useful for a variety of applications.

**Figure 14:**
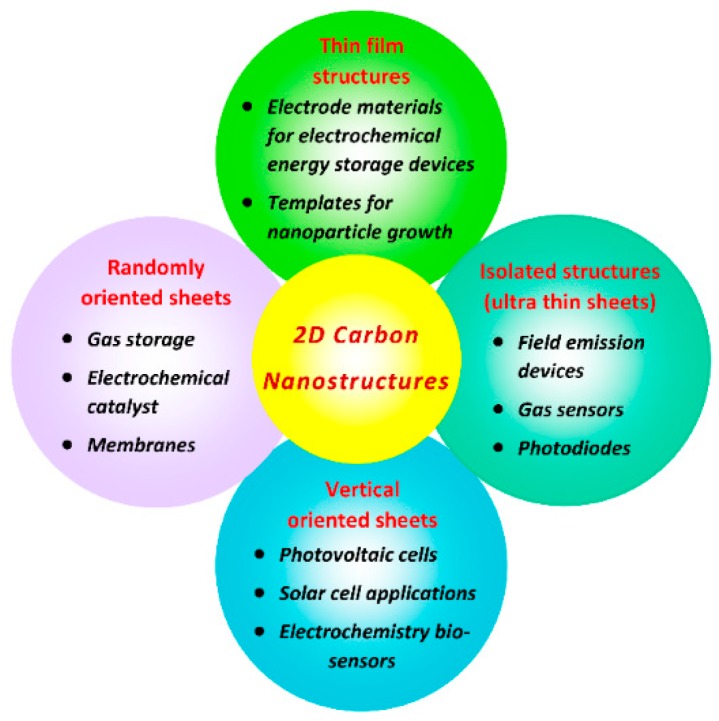
Schematic representations of possible applications of CNWs/other 2D carbon materials.

Opportunities of CNWs are the possibilities of applications. The major applications of CNWs in different fields are displayed in Figure 14. Considering the structure and morphology of CNWs, the large surface area of CNWs is one of the main advantages, which can be widely used for energy device applications and gas storage devices. The vertically-oriented structure with interlayer spacings can be a suitable material for the catalyst supporter in the fuel cells. Highly branched CNWs with a large surface to volume ratio is a potential material for gas sensing applications. Exfoliation of CNWs from the substrate can open up possibilities for using them as membrane filters. Plasma-surface treatment with different gases is useful for modifying the properties of CNWs for specific applications. Sharp edges with the high crystalline nature of CNWs are suitable for field emission applications. F-terminated CNWs with higher stability against degradation are suitable for hydrophobic coatings, which can be achieved through either fluorine-containing plasma treatment or growth from FC precursors. Nitrogen plasma-treatment can produce N-doped CNWs with improved electrical properties and capacitance. The maximum percentage of N-doping in CNWs so far reported is approximately 25%, which can be useful for various electrical and electrochemical applications. Production of CNWs from the nitrogen-containing precursor (e.g., aromatic precursor contains amino groups) can be an alternative method to enhance the concentration of nitrogen both in interstitial and edges of CNWs. Oxygenated CNWs can become able to produce a PL effect, applicable for designing optoelectronic devices. Hydrogenation or surface functionalization with hydrogen plasma enhances the hydrophilic properties of CNWs, which enhances the reaction towards electrolytes in electrochemical energy storage devices to provide better results. Moreover, this hydrophilic nature promotes their applications in biosensing, energy storage devices, and catalytic reactions. The intrinsic electrical property (without any functional groups/dopants) of CNWs is not yet revealed; investigating the intrinsic behavior of CNWs will be a great step for the next generation of electronic devices.

## 9. Conclusions

The open boundary structure, vertical alignment with interlayer spacing, and high crystallinity of 2D CNWs has extended carbon-related research into various areas. Employing different gaseous and aromatic precursors to a PECVD system, which are activated by different plasma sources, has successfully demonstrated the synthesis of vertically-oriented CNWs. There have been many studies to identify the parameters responsible for the growth; starting from the precursor gas, the mode of plasma coupling, plasma power source, the pressure inside the system, duration of growth, and electric field to substrate temperature. All these were systematically presented in this paper. In addition to this, the major advantage of plasma synthesis is the production of highly pure 2D carbon materials in a rather controllable manner. However, the responsible plasma species and growth mechanisms still have gaps to be deeply explored. Moreover, the future challenges in CNW research are also connected with: The control of the height uniformity with large interlayer spacing and higher crystallinity; bridging the low growth rates for upscaling; precise isolation of single nanowalls; and uniform doping of nitrogen on CNW lattices with higher concentration.

## Figures and Tables

**Table 1:** Overview of plasma-enhanced syntheses for different two-dimensional (2D) carbon nanostructures *.

Plasma Source	Source Gas	Parameters					Nanostructure and Characteristics	Ref.
		Temperature (°C)	Pressure (Torr)	Flow Rate (sccm)	Growth Time (min)	Power (W)		
**MW**	CH_4_:H_2_	650–700	1	40:10	8–10	500	CNW, Uniformly oriented carbon sheets	[3]
	acetylene, ammonia	High	10	Flow ratio <1	10	500	CNW, Grape like and aggregate structure	[24]
	H_2_, CH_4_	-	1.7	80:20 and 80: 5	0.17–15	500	CNWs with a higher growth rate	[25]
	CH_4_/H_2_	700	40	200 sccm Ratio: 1:8	1–50	2000	FLG, vertically-aligned sheets with thickness 4–6 atomic layers	[26]
	CH_4_/H_2_	350	2.2 × 10^−3^	-	10	1300	CNWs with 660 nm height	[51]
	CH_4_, H_2_, Ar	100–400	<1 Torr	30, 50, 20	1–2	16,000	Graphene sheets, A continuous graphene film with 294 mm width	[27]
	C_2_H_2_:Ar	240	<1 Torr	10, 200	2–4	1200	Few layer graphene sheets with pacing 0.345 nm	[28]
	CH_4_/H_2_	450–700	20	80:1	1	1400	High-quality centimeter scale graphene sheet	[48]
	He, H_2_, CH_4_	680	atm	1, 25, 25	30	350	Vertically grown carbon nanowall	[31]
**RFICP**	CH_4_, H_2_/Ar	Low	1	1100	0–60	600	High-quality graphene layers with significant growth kinetics	[52]
	CH_4_/H_2_	680	90 mTorr	0:100–95:5	-	900	Free-standing sub-nanometer graphite sheets	[53]
	CH_4_, Ar	700–850	10–60 mTorr	7, 1.4	30–60	500	The growth of carbon nanowalls	[45]
	Al(acac)_3_, Ar	350, 425, 500	8 Pa	1.66, 40	50	500	CNWs with different structures	[50]
**RFCCP**	C_2_F_6_, CH_4_, CHF_3_, C_4_F_6_, and H_2_	500	100 mTorr	Carbon precursor: 15, and 30	3–8 h	400	Vertical growth of carbon nanowalls	[41]
	C_2_F_6_, H_2_ (Radical injection)	600	0.1–1.2	50, 100	30 Min–10 h	MW/VHF 250/300	The highly reliable growth of carbon nanowall	[42]
	C_2_F_6_, H_2_	580	1.2	50, 100	30 s to 60 min	MW/VHF 250/270	Vertically standing CNWs with a uniform height	[49]
**RF plasma beam**	C_2_H_2_, H_2_, Ar	700	00075–2.25	1–20, 1–20, 100–1000	60	50–500	CNWs with large surface area and sharp edges	[54]
	Ar, H_2_, C_2_H_2_	200–700	1	1050, 25, 1	60	300	Various nanostructures including CNWs	[55]
**EBEP**	CH_4_/H_2_	570	10–30 mTorr	-	10–90	Electron-voltage ~60–100 V	Vertically aligned well definite CNW	[45]
**DC**	Ar:H_2_, CH_4_, Ar	700	atm	1000, 0.15, 1.35	30 s–10	0–10 kV	CNWs	[56]

* Al(acac)_3_: Aluminium acetylacetonate, MW: Microwave, FLG: Few-layer graphene sheet, RFICP: Radio-frequency inductively coupled plasma, RFCCP: Radio-frequency capacitively coupled plasma, RF: Radio frequency, EBEP: Electron beam excited plasma, DC: Direct current, VHF: Very high frequency.

**Table 2:** Overview of different gases and plasma sources used for the synthesis of 2D carbon nanostructures and main influence of radical species *.

Precursor	Flow Rate (sccm)	Plasma Source	Structure and Properties	Effect of Radical Species
**CH_4_:H_2_**	20:80 & 5:80	MWPECVD	CNWs with higher growth rate with thickness 20 nm	Hydrogen radicals help the plasma ignition and enhance the growth rate by higher carbon dimer density [25]
	200 sccm Ratio: 1:8	MWPECVD	FLG, vertically aligned sheets with thickness 4–6 atomic layers	The average dimensions of the flakes reduce with increase in hydrogen flow compared to CH_4_ flow rate [26]
	1:80, 1:40, 1:10	MWPECVD	High-quality centimeter scale graphene sheet	The flow of more CH_4_ in a ratio of 80:1 leads to the production of high-quality graphene monolayer without defects [48]
	0:100–95:5	ICPECVD	Free-standing sub-nanometer graphite sheets	H radicals help with producing carbon nanosheets with thickness 1 nm with an average height of 250 nm [53]
	-	EBEPECVD	Vertically aligned well definite CNW	The height of the CNW increased by 3 times and spacing between individual layers increased by 5–10 times [45]
**CH_4_,** **H_2_,** **Ar**	3–7.5, 12.4, 10–50	ICPECVD	Ordered carbon nanostructures	The electron density growth influenced by the rise of argon density [72]
**CH_4_ & Ar:H_2_**	1 & 100 (90:10)	ICPECVD	High-quality graphene layers with significant growth kinetics	A single layer of graphene sheets formed due to the high H radical density with help to etch C atoms [52]
**Ar:H_2_,** **CH_4_: Ar,** **OH radicals**	1000, 0.15:1.35	DC-atm	CNWs	OH radicals effectively remove a-C, increases CNW crystallinity and enhance the initial nucleation process [56]
**CH_4_, Ar**	7, 1.4	ICPECVD	The growth of carbon nanowalls	H and Ar radical helps to remove the amorphous carbon and CNW with a smooth surface, saturated morphology and thickness grown [31]
**C_2_F_6_, CH_4_, CHF_3_, C_4_F_6,_ and H_2_**	15, and 30	CCPECVD	Vertical growth of carbon nanowalls	Injection of H radicals to the fluorocarbon radicals produce *sp*^2^ bonds on the surface and form a continuous network to form CNW [41]
**C_2_F_6_,** **H_2_,** **O_2_**	50, 100, 256	CCPECVD	The highly reliable growth of carbon nanowall	O_2_ plasma chamber cleaning increases the growth of CNWs with good reproducibility [42]
**C_2_F_6_,** **H_2_,** **O_2_**	50, 100, 5	CCPECVD (Radical injection)	Vertically standing CNWs with a uniform height	O_2_ influence the effective removal of amorphous carbon from the CNW surface and controlling the nucleation [49]
**H_2_, C_2_H_2_:NH_3_**	100, Ratio < 1	DCPECVD	Vertically aligned carbon nanostructures	The high amount of NH_3_ in the system increases the etching rate by producing large H radicals and remove amorphous carbon between the structures [73]

* MWPECVD: Microwave plasma-enhanced chemical vapor deposition, ICPECVD: Inductively coupled plasma-enhanced chemical vapor deposition, EBEPECVD: Electron beam excited plasma-enhanced chemical vapor deposition, CCPECVD: Capacitively coupled plasma-enhanced chemical vapor deposition, DCPECVD: Direct current plasma-enhanced chemical vapor deposition.

**Table 3:** Different plasma treatments on 2D carbon nanostructures and observed modifications.

Plasma Treatment	Morphology	Changes in the Structure/Property	Ref.
**Hydrogen**	FLGs	Few layers of highly crystalline graphene sheets with few defects	[26]
	Petal-like nanosheets	Carbon nanosheets from CNTs with a thickness 300 to 500 nm	[99]
	CNWs	Large area free standing CNWs in a well-isolated manner	[112]
		Thin CNW films with ultra-low total reflectance (0.13%) for black body coating	[113]
		Controllable high-quality growth with good electric performance	[114]
	Few-layer graphene	Semiconducting sheets with one magnitude less carrier mobility and two order increase in sheet resistance	[115]
	Thin layer graphene sheets	Defects due to hydrogen plasma make fluctuations in optical properties	[116]
	CNWs	A surface roughened layer formed with a higher surface area	[101]
**Oxygen**	Thin carbon films	Re-structured carbon coatings with higher scratch resistance	[117]
	CNWs	Alters the adhesive macrophage properties	[118]
	CNWs	Increasing the surface activity of CNWs to act as a template for fabricating nanostructured materials	[119]
	CNWs	O_2_ plasma addition effectively increases the graphitization of carbon nanowalls and controlling the nucleation of CNW growth	[49]
	Few graphene sheets	Enhancing the *p*-type semiconducting behaviors of graphene nanosheets with strong photoluminescence effect	[120]
	Multilayer graphene sheets	The exponential decrease of conductance and transconductance	[121]
	Graphene sheets	Transformation of semi-metallic nature of graphene sheets into semiconducting via the opening of a band gap	[103]
**Argon**	Single layer graphene	Increases the photoresponse by Ar plasma-induced defects	[122]
	Graphene paper	Reduced the aggregation and forms surface protrusions and improves field emission properties	[123]
	CNW	Induce dangling bonds on the surface and resulting in the formation of nucleation sites	[124]
	GNWs	Field emission properties of GNWs increases by improving graphitic order and removing impurities	[17]
	CNWs	Continuous change in the morphology from 1D to 2D with an increase in Ar flow	[54]
	FLGs	Field emission properties enhanced by forming sharp edges and defects	[125]
**Nitrogen**	CNWs	Electrical conductance of N-doped CNWs increases compared to undoped CNWs, N-doped CNWs exhibits *n*-type conduction, N act as donor	[126]
	Graphene sheets	Varying *p*-type conducting behavior to *n*-type with increasing N-concentration and possess spin-polarized band structure	[127]
	CNWs	*n*-type conductivity ranging from 1.9 to 330 Ω^−1^ cm^−1^ due to nitrogen incorporated defects in the amorphous carbon	[75]
	CNWs	The drastic increase in the electron emission current from the CNW edges treatment from 1 to 100 µA	[128]
	CNWs	Higher electrochemical reactivity for the N-doped CNWs	[129]
	CNWs	Maintain *p*-type conducting behavior and increases the carrier mobility	[130]
**Other plasmas**	Fluorination in the a-C	Hydrophobicity of a-C films enhanced by fluorinated plasma treatments	[131]
	Boron-doped graphene	The bandgap of the B-doped graphene widened to 0.54 eV from 0 eV	[109]
	Chlorinated graphene sheets	The large surface area with uniform morphology and possess a *p*-type semiconducting behavior	[132]
	Chlorinated graphene sheets	Reduces the sheet resistance and enhance optical transparency via C-Cl bonds	[111]

## References

[1] Kroto H.W., Heath J.R., O’Brien S.C., Curl R.F., Smalley R.E. (1985). C60: Buckminsterfullerene. Nature.

[2] Ando Y., Zhao X., Ohkohchi M. (1997). Production of petal-like graphite sheets by hydrogen arc discharge. Carbon N. Y..

[3] Wu Y., Qiao P., Chong T., Shen Z. (2002). Carbon nanowalls grown by microwave plasma enhanced chemical vapor deposition. Adv. Mater..

[4] Novoselov K.S., Geim A.K., Morozov S.V., Jiang D., Zhang Y., Dubonos S.V., Grigorieva I.V., Firsov A.A. (2004). Electric field in atomically thin carbon films. Science.

[5] Machino T., Takeuchi W., Kano H., Hiramatsu M., Hori M. (2009). Synthesis of platinum nanoparticles on two-dimensional carbon nanostructures with an ultrahigh aspect ratio employing supercritical fluid chemical vapor deposition process. Appl. Phys. Express.

[6] Giorgi L., Makris T.D., Giorgi R., Lisi N., Salernitano E. (2007). Electrochemical properties of carbon nanowalls synthesized by HF-CVD. Sensors Actuators B Chem..

[7] Lehmann K., Yurchenko O., Melke J., Fischer A., Urban G. (2018). High electrocatalytic activity of metal-free and non-doped hierarchical carbon nanowalls towards oxygen reduction reaction. Electrochim. Acta.

[8] Wu J., Shao Y., Wang B., Ostrikov K.K., Feng J., Cheng Q. (2016). Plasma-Produced Vertical Carbonous Nanoflakes for Li-Ion Batteries. Plasma Process. Polym..

[9] Tanaike O., Kitada N., Yoshimura H., Hatori H., Kojima K., Tachibana M. (2009). Lithium insertion behavior of carbon nanowalls by dc plasma CVD and its heat-treatment effect. Solid State Ionics.

[10] Krivchenko V.A., Itkis D.M., Evlashin S.A., Semenenko D.A., Goodilin E.A., Rakhimov A.T., Stepanov A.S., Suetin N.V., Pilevsky A.A., Voronin P.V. (2012). Carbon nanowalls decorated with silicon for lithium-ion batteries. Carbon N. Y..

[11] Dyakonov P., Mironovich K., Svyakhovskiy S., Voloshina O., Dagesyan S., Panchishin A., Suetin N., Bagratashvili V., Timashev P., Shirshin E. (2017). Carbon nanowalls as a platform for biological SERS studies. Sci. Rep..

[12] Yang B., Wu Y., Zong B., Shen Z. (2002). Electrochemical Synthesis and Characterization of Magnetic Nanoparticles on Carbon Nanowall Templates. Nano Lett..

[13] Hou K., Outlaw R.A., Wang S., Zhu M., Quinlan R.A., Manos D.M., Kordesch M.E., Arp U., Holloway B.C. (2008). Uniform and enhanced field emission from chromium oxide coated carbon nanosheets. Appl. Phys. Lett..

[14] Yu K., Bo Z., Lu G., Mao S., Cui S., Zhu Y., Chen X., Ruoff R.S., Chen J. (2011). Growth of carbon nanowalls at atmospheric pressure for one-step gas sensor fabrication. Nanoscale Res. Lett..

[15] Russo P., Xiao M., Zhou N.Y. Carbon nanowalls: A new material for resistive switching memory devices. Carbon N. Y..

[16] Wang J., Ito T. (2007). CVD growth and field emission characteristics of nano-structured films composed of vertically standing and mutually intersecting nano-carbon sheets. Diam. Relat. Mater..

[17] Hojati-Talemi P., Simon G.P. (2011). Field emission study of graphene nanowalls prepared by microwave-plasma method. Carbon N. Y..

[18] Sankaran K.J., Ficek M., Kunuku S., Panda K., Yeh C.J., Park J.Y., Sawczak M., Michałowski P.P., Leou K.C., Bogdanowicz R. (2018). Self-organized multi-layered graphene-boron-doped diamond hybrid nanowalls for high-performance electron emission devices. Nanoscale.

[19] Vizireanu S., Ionita M.D., Dinescu G., Enculescu I., Baibarac M., Baltog I. (2012). Post-synthesis carbon nanowalls transformation under hydrogen, oxygen, nitrogen, tetrafluoroethane and sulfur hexafluoride plasma treatments. Plasma Process. Polym..

[20] Segundo E.H., Fontana L.C., Recco A.A.C., Scholtz J.S., Nespolo Vomstein M.A., Becker D. (2018). Graphene nanosheets obtained through graphite powder exfoliation in pulsed underwater electrical discharge. Mater. Chem. Phys..

[21] Chen Z., Guo X., Zhu L., Li L., Liu Y., Zhao L., Zhang W., Chen J., Zhang Y., Zhao Y. (2018). Direct growth of graphene on vertically standing glass by a metal-free chemical vapor deposition method. J. Mater. Sci. Technol..

[22] Chen J., Guo Y., Wen Y., Huang L., Xue Y., Geng D., Wu B., Luo B., Yu G., Liu Y. (2013). Two-stage metal-catalyst-free growth of high-quality polycrystalline graphene films on silicon nitride substrates. Adv. Mater..

[23] Hwang J., Kim M., Campbell D., Alsalman H.A., Kwak J.Y., Shivaraman S., Woll A.R., Singh A.K., Hennig R.G., Gorantla S. (2013). Van der waals epitaxial growth of graphene on sapphire by chemical vapor deposition without a metal catalyst. ACS Nano.

[24] Chuang A.T.H., Robertson J., Boskovic B.O., Koziol K.K.K. (2007). Three-dimensional carbon nanowall structures. Appl. Phys. Lett..

[25] Tanaka K., Yoshimura M., Okamoto A., Ueda K. (2005). Growth of carbon nanowalls on a SiO2 substrate by microwave plasma-enhanced chemical vapor deposition. Jpn. J. Appl. Phys. Part 1 Regul. Pap. Short Notes Rev. Pap..

[26] Malesevic A., Vitchev R., Schouteden K., Volodin A., Zhang L., Van Tendeloo G., Vanhulsel A., Van Haesendonck C. (2008). Synthesis of few-layer graphene via microwave plasma-enhanced chemical vapour deposition. Nanotechnology.

[27] Yamada T., Ishihara M., Kim J., Hasegawa M., Iijima S. (2012). A roll-to-roll microwave plasma chemical vapor deposition process for the production of 294mm width graphene films at low temperature. Carbon.

[28] Kalita G., Wakita K., Umeno M. (2012). Low temperature growth of graphene film by microwave assisted surface wave plasma CVD for transparent electrode application. RSC Adv..

[29] Bo Z., Yang Y., Chen J., Yu K., Yan J., Cen K. (2013). Plasma-enhanced chemical vapor deposition synthesis of vertically oriented graphene nanosheets. Nanoscale.

[30] Zhong Y.L., Tian Z., Simon G.P., Li D. (2015). Scalable production of graphene via wet chemistry: Progress and challenges. Mater. Today.

[31] Hiramatsu M., Hori M. (2010). Carbon Nanowalls: Synthesis and Emerging Applications.

[32] Woehrl N., Ochedowski O., Gottlieb S., Shibasaki K., Schulz S. (2014). Plasma-enhanced chemical vapor deposition of graphene on copper substrates. AIP Adv..

[33] Lieberman M.A., Lichtenberg A.J. (2005). Principles of Plasma Discharges and Materials Processing: Second Edition.

[34] Wang Q., Wang X., Chai Z., Hu W. (2013). Low-temperature plasma synthesis of carbon nanotubes and graphene based materials and their fuel cell applications. Chem. Soc. Rev..

[35] Conrads H., Schmidt M. (2000). Plasma generation and plasma sources. Plasma Sources Sci. Technol..

[36] Azarenkov N.A., Denisenko I.B., Ostrikov K.N. (1995). A model of a large-area planar plasma producer based on surface wave propagation in a plasma-metal structure with a dielectric sheath. J. Phys. D Appl. Phys..

[37] Stratakos Y., Zeniou A., Gogolides E. (2017). Comparison of Helical and Helicon Antennas as Sources of Plasma Excitation Using a Full Wave 3D Electromagnetic Analysis in Vacuum. Plasma Process. Polym..

[38] Cheng Q., Xu S., Ostrikov K. (2010). (Ken) Single-step, rapid low-temperature synthesis of Si quantum dots embedded in an amorphous SiC matrix in high-density reactive plasmas. Acta Mater..

[39] Li M., Liu D., Wei D., Song X., Wei D., Wee A.T.S. (2016). Controllable Synthesis of Graphene by Plasma-Enhanced Chemical Vapor Deposition and Its Related Applications. Adv. Sci..

[40] Hertl M., Jolly J., Baravian G. (2002). Detection of hydrogen atoms in SiH4-H2radio-frequency plasmas using two-photon laser-induced fluorescence. J. Appl. Phys..

[41] Hiramatsu M., Shiji K., Amano H., Hori M. (2004). Fabrication of vertically aligned carbon nanowalls using capacitively coupled plasma-enhanced chemical vapor deposition assisted by hydrogen radical injection. Appl. Phys. Lett..

[42] Kondo S., Hori M., Yamakawa K., Den S., Kano H., Hiramatsu M. (2008). Highly reliable growth process of carbon nanowalls using radical injection plasma-enhanced chemical vapor deposition. J. Vac. Sci. Technol. B Microelectron. Nanom. Struct..

[43] Banerjee D., Mukherjee S., Chattopadhyay K.K. (2011). Synthesis of amorphous carbon nanowalls by DC-PECVD on different substrates and study of its field emission properties. Appl. Surf. Sci..

[44] Hara T., Hamagaki M., Sanda A., Aoyagi Y., Namba S. (1986). New high current low energy ion source. Jpn. J. Appl. Phys..

[45] Mori T., Hiramatsu M., Yamakawa K., Takeda K., Hori M. (2008). Fabrication of carbon nanowalls using electron beam excited plasma-enhanced chemical vapor deposition. Diam. Relat. Mater..

[46] Levchenko I., Cvelbar U., Keidar M. (2016). Graphene Flakes in Arc Plasma: Conditions for the Fast Single-Layer Growth. Graphene.

[47] Keidar M., Shashurin A., Li J., Volotskova O., Kundrapu M., Zhuang T. (2011). Sen Arc plasma synthesis of carbon nanostructures: Where is the frontier?. J. Phys. D Appl. Phys..

[48] Kim Y., Song W., Lee S.Y., Jeon C., Jung W., Kim M., Park C.Y. (2011). Low-temperature synthesis of graphene on nickel foil by microwave plasma chemical vapor deposition. Appl. Phys. Lett..

[49] Kondo S., Kawai S., Takeuchi W., Yamakawa K., Den S., Kano H., Hiramatsu M., Hori M. (2009). Initial growth process of carbon nanowalls synthesized by radical injection plasma-enhanced chemical vapor deposition. J. Appl. Phys..

[50] Giese A., Schipporeit S., Buck V., Wöhrl N. (2018). Synthesis of carbon nanowalls from a single-source metal-organic precursor. Beilstein J. Nanotechnol..

[51] Park J.K., Kang H., Kim J.H., Choi W. (2018). Improvement of Electrical Properties of Carbon Nanowall by the Deposition of Thin Film. J. Nanosci. Nanotechnol..

[52] Van Nang L., Kim E.-T. (2012). Controllable Synthesis of High-Quality Graphene Using Inductively-Coupled Plasma Chemical Vapor Deposition. J. Electrochem. Soc..

[53] Wang J.J., Zhu M.Y., Outlaw R.A., Zhao X., Manos D.M., Holloway B.C., Mammana V.P. (2004). Free-standing subnanometer qraphite sheets. Appl. Phys. Lett..

[54] Vizireanu S., Nistor L., Haupt M., Katzenmaier V., Oehr C., Dinescu G. (2008). Carbon nanowalls growth by radiofrequency plasma-beam-enhanced chemical vapor deposition. Plasma Process. Polym..

[55] Acosta Gentoiu M., Betancourt-Riera R., Vizireanu S., Burducea I., Marascu V., Stoica S.D., Bita B.I., Dinescu G., Riera R. (2017). Morphology, Microstructure, and Hydrogen Content of Carbon Nanostructures Obtained by PECVD at Various Temperatures. J. Nanomater..

[56] Bo Z., Yu K., Lu G., Wang P., Mao S., Chen J. (2011). Understanding growth of carbon nanowalls at atmospheric pressure using normal glow discharge plasma-enhanced chemical vapor deposition. Carbon N. Y..

[57] Lehmann K., Yurchenko O., Urban G. (2016). Effect of the aromatic precursor flow rate on the morphology and properties of carbon nanostructures in plasma enhanced chemical vapor deposition. RSC Adv..

[58] Bundaleska N., Henriques J., Abrashev M., Botelho do Rego A.M., Ferraria A.M., Almeida A., Dias F.M., Valcheva E., Arnaudov B., Upadhyay K.K. (2018). Large-scale synthesis of free-standing N-doped graphene using microwave plasma. Sci. Rep..

[59] Seo D.H., Rider A.E., Kumar S., Randeniya L.K., Ostrikov K. (2013). Vertical graphene gas- and bio-sensors via catalyst-free, reactive plasma reforming of natural honey. Carbon N. Y..

[60] Seo D.H., Pineda S., Yick S., Bell J., Han Z.J., Ostrikov K. (2015). Plasma-enabled sustainable elemental lifecycles: Honeycomb-derived graphenes for next-generation biosensors and supercapacitors. Green Chem..

[61] Zhao J., Shaygan M., Eckert J., Meyyappan M., Rümmeli M.H. (2014). A growth mechanism for free-standing vertical graphene. Nano Lett..

[62] Bo Z., Mao S., Jun Han Z., Cen K., Chen J., Ostrikov K. (2015). Emerging energy and environmental applications of vertically-oriented graphenes. Chem. Soc. Rev..

[63] Zhu M., Wang J., Holloway B.C., Outlaw R.A., Zhao X., Hou K., Shutthanandan V., Manos D.M. (2007). A mechanism for carbon nanosheet formation. Carbon N. Y..

[64] Hiramatsu M., Hori M. (2006). Fabrication of carbon nanowalls using novel plasma processing. Jpn. J. Appl. Phys. Part 1 Regul. Pap. Short Notes Rev. Pap..

[65] Baranov O., Levchenko I., Xu S., Lim J.W.M., Cvelbar U., Bazaka K. (2018). Formation of vertically oriented graphenes: what are the key drivers of growth?. 2D Mater..

[66] Davami K., Shaygan M., Kheirabi N., Zhao J., Kovalenko D.A., Rümmeli M.H., Opitz J., Cuniberti G., Lee J.S., Meyyappan M. (2014). Synthesis and characterization of carbon nanowalls on different substrates by radio frequency plasma enhanced chemical vapor deposition. Carbon N. Y..

[67] Denysenko I., Ostrikov K., Yu M.Y., Azarenkov N.A. (2007). Effects of ions and atomic hydrogen in plasma-assisted growth of single-walled carbon nanotubes. J. Appl. Phys..

[68] Nozaki T., Goto T., Okazaki K., Ohnishi K., Mangolini L., Heberlein J., Kortshagen U. (2006). Deposition of vertically oriented carbon nanofibers in atmospheric pressure radio frequency discharge. J. Appl. Phys..

[69] Ostrikov K. (2011). Nanoscale transfer of energy and matter in plasma-surface interactions. IEEE Trans. Plasma Sci..

[70] Mao M., Bogaerts A. (2010). Investigating the plasma chemistry for the synthesis of carbon nanotubes/nanofibres in an inductively coupled plasma enhanced CVD system: The effect of different gas mixtures. J. Phys. D Appl. Phys..

[71] Chhowalla M., Teo K.B.K., Ducati C., Rupesinghe N.L., Amaratunga G.A.J., Ferrari A.C., Roy D., Robertson J., Milne W.I. (2001). Growth process conditions of vertically aligned carbon nanotubes using plasma enhanced chemical vapor deposition. J. Appl. Phys..

[72] Denysenko I.B., Xu S., Long J.D., Rutkevych P.P., Azarenkov N.A., Ostrikov K. (2004). Inductively coupled Ar/CH4/H2 plasmas for low-temperature deposition of ordered carbon nanostructures. J. Appl. Phys..

[73] Chuang A.T.H., Boskovic B.O., Robertson J. (2006). Freestanding carbon nanowalls by microwave plasma-enhanced chemical vapour deposition. Diam. Relat. Mater..

[74] Mantzaris N.V., Gogolides E., Boudouvis A.G., Rhallabi A., Turban G. (1996). Surface and plasma simulation of deposition processes: CH4plasmas for the growth of diamondlike carbon. J. Appl. Phys..

[75] Teii K., Shimada S., Nakashima M., Chuang A.T.H. (2009). Synthesis and electrical characterization of n -type carbon nanowalls. J. Appl. Phys..

[76] Vizireanu S., Stoica S.D., Luculescu C., Nistor L.C., Mitu B., Dinescu G. (2010). Plasma techniques for nanostructured carbon materials synthesis. A case study: Carbon nanowall growth by low pressure expanding RF plasma. Plasma Sources Sci. Technol..

[77] Zhu M.Y., Outlaw R.A., Bagge-Hansen M., Chen H.J., Manos D.M. (2011). Enhanced field emission of vertically oriented carbon nanosheets synthesized by C2H2/H2 plasma enhanced CVD. Carbon N. Y..

[78] Hiramatsu M., Kato K., Lau C.H., Foord J.S., Hori M. (2003). Measurement of C2radical density in microwave methane/hydrogen plasma used for nanocrystalline diamond film formation. Diam. Relat. Mater..

[79] Shiomi T., Nagai H., Kato K., Hiramatsu M., Nawata M. (2001). Detection of C2 radicals in low-pressure inductively coupled plasma source for diamond chemical vapor deposition. Diam. Relat. Mater..

[80] Hori M., Kondo H., Hiramatsu M. (2011). Radical-controlled plasma processing for nanofabrication. J. Phys. D Appl. Phys..

[81] Shiji K., Hiramatsu M., Enomoto A., Nakamura M., Amano H., Hori M. (2005). Vertical growth of carbon nanowalls using rf plasma-enhanced chemical vapor deposition. Diam. Relat. Mater..

[82] Shang N.G., Au F.C.K., Meng X.M., Lee C.S., Bello I., Lee S.T. (2002). Uniform carbon nanoflake films and their field emissions. Chem. Phys. Lett..

[83] Chatei H., Belmahi M., Assouar M.B., Le Brizoual L., Bourson P., Bougdira J. (2006). Growth and characterisation of carbon nanostructures obtained by MPACVD system using CH4/CO2gas mixture. Diam. Relat. Mater..

[84] Wang J., Zhu M., Outlaw R.A., Zhao X., Manos D.M., Holloway B.C. (2004). Synthesis of carbon nanosheets by inductively coupled radio-frequency plasma enhanced chemical vapor deposition. Carbon N. Y..

[85] Wu Y., Yang B., Zong B., Sun H., Shen Z., Feng Y. (2004). Carbon nanowalls and related materials. J. Mater. Chem..

[86] Kondo H., Hori M., Hiramatsu M. (2011). Nucleation and Vertical Growth of Nano-Graphene Sheets. Graphene—Synthesis, Characterization, Properties and Applications.

[87] Suzuki S., Chatterjee A., Cheng C.L., Yoshimura M. (2011). Effect of hydrogen on carbon nanowall growth by microwave plasma-enhanced chemical vapor deposition. Jpn. J. Appl. Phys..

[88] Takeuchi W., Hiramatsu M., Tokuda Y., Kano H., Hori M. Control of Structure and Electrical properties of Carbon Nanowalls: Effect of N_2_/O_2_ addition to fluorocarbon plasma CVD with H radical injection. Proceedings of the 2008 International Conference on Solid State Devices and Materials.

[89] Paschen F. (1889). Ueber die zum Funkenübergang in Luft, Wasserstoff und Kohlensäure bei verschiedenen Drucken erforderliche Potentialdifferenz. Ann. Phys..

[90] Hiramatsu M., Tomatsu M., Kondo H., Hori M. Synthesis of vertical nanographene network as platform for electrochemical applications. Proceedings of the 2017 World Congress on Advances in Nano, Bio, Robotics and Energy (ANBRE17).

[91] Takeuchi W., Sasaki H., Kato S., Takashima S., Hiramatsu M., Hori M. (2009). Development of measurement technique for carbon atoms employing vacuum ultraviolet absorption spectroscopy with a microdischarge hollow-cathode lamp and its application to diagnostics of nanographene sheet material formation plasmas. J. Appl. Phys..

[92] Ostrikov K., Neyts E.C., Meyyappan M. (2013). Plasma nanoscience: From nano-solids in plasmas to nano-plasmas in solids. Adv. Phys..

[93] Yang C., Bi H., Wan D., Huang F., Xie X., Jiang M. (2013). Direct PECVD growth of vertically erected graphene walls on dielectric substrates as excellent multifunctional electrodes. J. Mater. Chem. A.

[94] Wiesemann K. (2014). A Short Introduction to Plasma Physics. arXiv.

[95] Kersten H., Deutsch H., Steffen H., Kroesen G.M.W., Hippler R. (2001). The energy balance at substrate surfaces during plasma processing. Vacuum.

[96] Krivchenko V., Shevnin P., Pilevsky A., Egorov A., Suetin N., Sen V., Evlashin S., Rakhimov A. (2012). Influence of the growth temperature on structural and electron field emission properties of carbon nanowall/nanotube films synthesized by catalyst-free PECVD. J. Mater. Chem..

[97] Kim J., Ishihara M., Koga Y., Tsugawa K., Hasegawa M., Iijima S. (2011). Low-temperature synthesis of large-area graphene-based transparent conductive films using surface wave plasma chemical vapor deposition. Appl. Phys. Lett..

[98] Kim S.Y., Choi W.S., Lee J.H., Hong B. (2014). Substrate temperature effect on the growth of carbon nanowalls synthesized via microwave PECVD. Mater. Res. Bull..

[99] Zeng L., Lei D., Wang W., Liang J., Wang Z., Yao N., Zhang B. (2008). Preparation of carbon nanosheets deposited on carbon nanotubes by microwave plasma-enhanced chemical vapor deposition method. Appl. Surf. Sci..

[100] Felten A., Bittencourt C., Colomer J.F., Van Tendeloo G., Pireaux J.J. (2007). Nucleation of metal clusters on plasma treated multi wall carbon nanotubes. Carbon N. Y..

[101] Jiang N., Wang H.X., Zhang H., Sasaoka H., Nishimura K. (2010). Characterization and surface modification of carbon nanowalls. J. Mater. Chem..

[102] Xu T., Yang J., Liu J., Fu Q. (2007). Surface modification of multi-walled carbon nanotubes by O2plasma. Appl. Surf. Sci..

[103] Nourbakhsh A., Cantoro M., Vosch T., Pourtois G., Clemente F., Van Der Veen M.H., Hofkens J., Heyns M.M., De Gendt S., Sels B.F. (2010). Bandgap opening in oxygen plasma-treated graphene. Nanotechnology.

[104] Dias A., Bundaleski N., Tatarova E., Dias F.M., Abrashev M., Cvelbar U., Teodoro O.M.N.D., Henriques J. (2016). Production of N-graphene by microwave N2-Ar plasma. J. Phys. D Appl. Phys..

[105] Stancu E.C., Stanciuc A.M., Vizireanu S., Luculescu C., Moldovan L., Achour A., Dinescu G. (2014). Plasma functionalization of carbon nanowalls and its effect on attachment of fibroblast-like cells. J. Phys. D Appl. Phys..

[106] Watanabe H., Kondo H., Hiramatsu M., Sekine M., Kumar S., Ostrikov K., Hori M. (2013). Surface chemical modification of carbon nanowalls for wide-range control of surface wettability. Plasma Process. Polym..

[107] Zhu L., Xiu Y., Xu J., Tamirisa P.A., Hess D.W., Wong C.P. (2005). Superhydrophobicity on two-tier rough surfaces fabricated by controlled growth of aligned carbon nanotube arrays coated with fluorocarbon. Langmuir.

[108] Lu C., Dong Q., Tulugan K., Park Y.M., More M.A., Kim J., Kim T.G. (2016). Characteristic Study of Boron Doped Carbon Nanowalls Films Deposited by Microwave Plasma Enhanced Chemical Vapor Deposition. J. Nanosci. Nanotechnol..

[109] Tang Y.B., Yin L.C., Yang Y., Bo X.H., Cao Y.L., Wang H.E., Zhang W.J., Bello I., Lee S.T., Cheng H.M. (2012). Tunable band gaps and p-type transport properties of boron-doped graphenes by controllable ion doping using reactive microwave plasma. ACS Nano.

[110] Sobaszek M., Siuzdak K., Ryl J., Sawczak M., Gupta S., Carrizosa S.B., Ficek M., Dec B., Darowicki K., Bogdanowicz R. (2017). Diamond Phase (*sp*^3^-C) Rich Boron-Doped Carbon Nanowalls (*sp*^2^-C): Physicochemical and Electrochemical Properties. J. Phys. Chem. C.

[111] Pham V.P., Kim K.H., Jeon M.H., Lee S.H., Kim K.N., Yeom G.Y. (2015). Low damage pre-doping on CVD graphene/Cu using a chlorine inductively coupled plasma. Carbon N. Y..

[112] Vizireanu S., Mitu B., Luculescu C.R., Nistor L.C., Dinescu G. (2012). PECVD synthesis of 2D nanostructured carbon material. Surf. Coatings Technol..

[113] Krivchenko V.A., Evlashin S.A., Mironovich K.V., Verbitskiy N.I., Nefedov A., Wöll C., Kozmenkova A.Y., Suetin N.V., Svyakhovskiy S.E., Vyalikh D.V. (2013). Carbon nanowalls: The next step for physical manifestation of the black body coating. Sci. Rep..

[114] Liu R., Chi Y., Fang L., Tang Z., Yi X. (2014). Synthesis of Carbon Nanowall by Plasma-Enhanced Chemical Vapor Deposition Method. J. Nanosci. Nanotechnol..

[115] Ryu S., Han M.Y., Maultzsch J., Heinz T.F., Kim P., Steigerwald M.L., Brus L.E. (2008). Reversible basal plane hydrogenation of graphene. Nano Lett..

[116] Eren B., Fu W., Marot L., Calame M., Steiner R., Meyer E. (2015). Spectroscopic ellipsometry on Si/SiO2/graphene tri-layer system exposed to downstream hydrogen plasma: Effects of hydrogenation and chemical sputtering. Appl. Phys. Lett..

[117] Guo M., Diao D., Fan X., Yang L., Yu L. (2014). Scratch behavior of re-structured carbon coating by oxygen plasma etching technology for magnetic disk application. Surf. Coat. Technol..

[118] Ion R., Vizireanu S., Stancu C.E., Luculescu C., Cimpean A., Dinescu G. (2015). Surface plasma functionalization influences macrophage behavior on carbon nanowalls. Mater. Sci. Eng. C.

[119] Wu Y.H., Yang B.J., Han G.C., Zong B.Y., Ni H.Q., Luo P., Chong T.C., Low T.S., Shen Z.X. (2002). Fabrication of a class of nanostructured materials using carbon nanowalls as the templates. Adv. Funct. Mater..

[120] Gokus T., Nair R.R., Bonetti A., Böhmler M., Lombardo A., Novoselov K.S., Geim A.K., Ferrari A.C., Hartschuh A. (2009). Making graphene luminescent by oxygen plasma treatment. ACS Nano.

[121] Kanghyun K., Hyung J.P., Woo B.C., Kook J.K., Gyu T.K., Wan S.Y. (2008). Electric property evolution of structurally defected multilayer grapheme. Nano Lett..

[122] Thiyagarajan K., Saravanakumar B., Kim S.J. (2015). Gate-tunable photoresponse of defective graphene: From ultraviolet to visible. ACS Appl. Mater. Interfaces.

[123] Liu J., Zeng B., Wu Z., Zhu J., Liu X. (2010). Improved field emission property of graphene paper by plasma treatment. Appl. Phys. Lett..

[124] Kondo S., Kondo H., Hiramatsu M., Sekine M., Hori M. (2010). Critical factors for nucleation and vertical growth of two dimensional nano-graphene sheets employing a novel Ar+beam with hydrogen and fluorocarbon radical injection. Appl. Phys. Express.

[125] Qi J.L., Wang X., Zheng W.T., Tian H.W., Hu C.Q., Peng Y.S. (2010). Ar plasma treatment on few layer graphene sheets for enhancing their field emission properties. J. Phys. D Appl. Phys..

[126] Takeuchi W., Ura M., Hiramatsu M., Tokuda Y., Kano H., Hori M. (2008). Electrical conduction control of carbon nanowalls. Appl. Phys. Lett..

[127] Wang C.D., Yuen M.F., Ng T.W., Jha S.K., Lu Z.Z., Kwok S.Y., Wong T.L., Yang X., Lee C.S., Lee S.T. (2012). Plasma-assisted growth and nitrogen doping of graphene films. Appl. Phys. Lett..

[128] Takeuchi W., Kondo H., Obayashi T., Hiramatsu M., Hori M. (2011). Electron field emission enhancement of carbon nanowalls by plasma surface nitridation. Appl. Phys. Lett..

[129] McClure J.P., Thornton J.D., Jiang R., Chu D., Cuomo J.J., Fedkiw P.S. (2012). Oxygen Reduction on Metal-Free Nitrogen-Doped Carbon Nanowall Electrodes. J. Electrochem. Soc..

[130] Cho H.J., Kondo H., Ishikawa K., Sekine M., Hiramatsu M., Hori M. (2014). Effects of nitrogen plasma post-treatment on electrical conduction of carbon nanowalls. Jpn. J. Appl. Phys..

[131] Zhou Y., Wang B., Zhang X., Zhao M., Li E., Yan H. (2009). The modifications of the surface wettability of amorphous carbon films. Colloids Surf. A Physicochem. Eng. Asp..

[132] Fan L., Zhang H., Zhang P., Sun X. (2015). One-step synthesis of chlorinated graphene by plasma enhanced chemical vapor deposition. Appl. Surf. Sci..

[133] Elias D.C., Nair R.R., Mohiuddin T.M.G., Morozov S.V., Blake P., Halsall M.P., Ferrari A.C., Boukhvalov D.W., Katsnelson M.I., Geim A.K. (2009). Control of Graphene’s Properties by Reversible Hydrogenation: Evidence for Graphane. Science.

[134] Watanabe H., Kondo H., Sekine M., Hiramatsu M., Hori M. (2012). Control of super hydrophobic and super hydrophilic surfaces of carbon nanowalls using atmospheric pressure plasma treatments. Jpn. J. Appl. Phys..

[135] Cui L., Chen J., Yang B., Sun D., Jiao T. (2015). RF-PECVD synthesis of carbon nanowalls and their field emission properties. Appl. Surf. Sci..

[136] Sahoo G., Polaki S.R., Ghosh S., Krishna N.G., Kamruddin M., Ostrikov K. (2018). (Ken) Plasma-tuneable oxygen functionalization of vertical graphenes enhance electrochemical capacitor performance. Energy Storage Mater..

[137] Sahoo G., Polaki S.R., Ghosh S., Krishna N.G., Kamruddin M. (2018). Temporal-stability of plasma functionalized vertical graphene electrodes for charge storage. J. Power Sources.

[138] Chen Q., Sun T., Song X., Ran Q., Yu C., Yang J., Feng H., Yu L., Wei D. (2017). Flexible electrochemical biosensors based on graphene nanowalls for the real-time measurement of lactate. Nanotechnology.

[139] Wang Y., Li J., Song K. (2014). Study on formation and photoluminescence of carbon nanowalls grown on silicon substrates by hot filament chemical vapor deposition. J. Lumin..

[140] Tatarova E., Dias A., Henriques J., Abrashev M., Bundaleska N., Kovacevic E., Bundaleski N., Cvelbar U., Valcheva E., Arnaudov B. (2017). Towards large-scale in free-standing graphene and N-graphene sheets. Sci. Rep..

[141] Chi Y.W., Hu C.C., Shen H.H., Huang K.P. (2016). New Approach for High-Voltage Electrical Double-Layer Capacitors Using Vertical Graphene Nanowalls with and without Nitrogen Doping. Nano Lett..

[142] Yen H.F., Horng Y.Y., Hu M.S., Yang W.H., Wen J.R., Ganguly A., Tai Y., Chen K.H., Chen L.C. (2015). Vertically aligned epitaxial graphene nanowalls with dominated nitrogen doping for superior supercapacitors. Carbon.

[143] Wang B.B., Cheng Q.J., Wang L.H., Zheng K., Ostrikov K. (2012). The effect of temperature on the mechanism of photoluminescence from plasma-nucleated, nitrogenated carbon nanotips. Carbon.

[144] Bundaleska N., Bundaleski N., Dias A., Dias F.M., Abrashev M., Filipič G., Cvelbar U., Rakočević Z., Kissovski Z., Henriques J. (2018). Microwave N_2_-Ar plasmas applied for N-graphene post synthesis. Mater. Res. Express.

